# Time-resolved RNA-seq analysis to unravel the *in vivo* competence induction by *Streptococcus pneumoniae* during pneumonia-derived sepsis

**DOI:** 10.1128/spectrum.03050-23

**Published:** 2024-02-02

**Authors:** Myung Whan Oh, Jingjun Lin, Sook Yin Chong, Shi Qian Lew, Tauqeer Alam, Gee W. Lau

**Affiliations:** 1 Department of Pathobiology, University of Illinois at Urbana-Champaign, Urbana, Illinois, USA; College of Dentistry, The Ohio State University, Columbus, Ohio, USA

**Keywords:** *Streptococcus pneumoniae*, pneumonia-derived sepsis, RNA-seq, competent state, host adaptation, Pht histidine triad proteins

## Abstract

Competence development in *Streptococcus pneumoniae* (pneumococcus) is tightly intertwined with virulence. In addition to genes encoding genetic transformation machinery, the competence regulon also regulates the expression of allolytic factors, bacteriocins, and cytotoxins. Pneumococcal competence system has been extensively interrogated *in vitro* where the short transient competent state upregulates the expression of three distinct phases of “early,” “late,” and “delayed” genes. Recently, we have demonstrated that the pneumococcal competent state develops naturally in mouse models of pneumonia-derived sepsis. To unravel the underlying adaptive mechanisms driving the development of the competent state, we conducted a time-resolved transcriptomic analysis guided by the spatiotemporal live *in vivo* imaging system of competence induction during pneumonia-derived sepsis. Mouse lungs infected by the serotype 2 strain D39 expressing a competent state-specific reporter gene (D39-*ssbB-luc*) were subjected to RNA sequencing guided by monitoring the competence development at 0, 12, 24, and, at the moribund state, >40 hours post-infection (hpi). Transcriptomic analysis revealed that the competence-specific gene expression patterns *in vivo* were distinct from those under *in vitro* conditions. There was significant upregulation of early, late, and some delayed phase competence-specific genes as early as 12 hpi, suggesting that the pneumococcal competence regulon is important for adaptation to the lung environment. Additionally, members of the histidine triad (*pht*) gene family were sharply upregulated at 12 hpi followed by a steep decline throughout the rest of the infection cycle, suggesting that Pht proteins participate in the early adaptation to the lung environment. Further analysis revealed that Pht proteins execute a metal ion-dependent regulatory role in competence induction.

**IMPORTANCE:**

The induction of pneumococcal competence for genetic transformation has been extensively studied *in vitro* but poorly understood during lung infection. We utilized a combination of live imaging and RNA sequencing to monitor the development of a competent state during acute pneumonia. Upregulation of competence-specific genes was observed as early as 12 hour post-infection, suggesting that the pneumococcal competence regulon plays an important role in adapting pneumococcus to the stressful lung environment. Among others, we report novel finding that the pneumococcal histidine triad (*pht*) family of genes participates in the adaptation to the lung environment and regulates pneumococcal competence induction.

## INTRODUCTION

*Streptococcus pneumoniae* (pneumococcus) is an opportunistic pathogen that colonizes the human nasopharyngeal tract. Despite being a commensal, pneumococcus has the capacity to cause serious diseases, including acute pneumonia, pneumonia-associated adverse cardiac event, invasive bacteremic sepsis, meningitis, and otitis media (1–3). Pneumococcus-mediated community-acquired pneumonia is a significant threat to young children, elderly, immunocompromised, and hospitalized patients (4, 5). The licensed capsular polysaccharide vaccine (Pneumovax) and pneumococcal conjugate vaccines have significantly alleviated disease burden and morbidity and mortality caused by pneumococcus although limited to vaccine-covered serotypes (6–8). The uniquely plastic nature of *S. pneumoniae* genome allows serotype switching and quick adaptations to increasing usage of antibiotic treatments hampering the eradication of pneumococcal diseases (9, 10).

During growth, pneumococcus naturally develops the competent state, during which exogenous DNA is acquired and integrated into the bacterial genome (9, 10). The pneumococcal competence system is heavily associated to virulence, biofilm formation, and antibiotic resistance. The natural competence is stimulated by the competence-stimulating peptide (CSP) encoded by the *comC* gene. The pre-CSP is processed and exported by the ComAB transporter and accumulates in the environment. When a threshold level is reached in a quorum sensing-dependent manner, CSP activates the ComDE two-component regulatory system for the initiation of the competence state.

Owing to the high-throughput sequencing technologies, an increasing number of transcriptomic analyses have been used to elucidate the pneumococcal competence development and host-pathogen interactions (11–15). In earlier microarray-based studies, Peterson et al. (16) and Dagkessamanskaia et al. (17) have laid the foundation for *in vitro* mechanistic investigations on competence development (18, 19) and for *in vivo* pneumococcal competence studies on competence-dependent colonization and virulence (20–26). While there are studies interrogating the pneumococcal competence regulon *in vitro* upon exposure to exogenously provided CSP (11, 16, 17) or in cultured lung epithelial cells (12), there is only one transcriptomic analysis that revealed the importance of pneumolysin-dependent competence induction in driving meningitis in a zebrafish (*Danio rerio*) disease model (15). Additionally, a time-resolved transcriptomic analysis attempting to decipher the *in vivo* pneumococcal competence regulon has never been reported.

Despite the extensive knowledge in the mechanisms of pneumococcal competence development *in vitro,* insights into host adaptation and key contributors to *in vivo* competence induction are lacking. Under *in vitro* conditions, competence-optimized pneumococcal strains (e.g., CP1200, CP1250, and R800) and the most widely studied serotype 2 pneumococcal strain D39 require specific culture conditions (e.g., C + Y medium) to enter the competent state (26–30). In contrast, D39 could only attain the competent state in the commonly used Todd Hewitt Broth with the provision of the CSP (26). The induction and duration of the competent state *in vitro* are transient, followed by a rapid shutoff approximately 40 minutes post-induction. Only a few studies have shown that pneumococcus naturally develops competence induction in living hosts (15, 20, 21, 26, 31, 32). By adopting the live *in vivo* imaging system (IVIS), we have previously demonstrated that D39 expressing the competent state-specific gene *ssbB* fused to the firefly luciferase (luc) reporter gene (D39-*ssbB-luc*), which does not enter the competent state naturally *in vitro*, could enter a prolonged competent state in mouse lung during acute pneumonia (26). D39-*ssbB-luc* was observed to enter the competent state between 20 and 24 hours post-infection (hpi), followed by the breach of alveolar-capillary barrier and systemic sepsis. Interestingly, the kinetics of initiating the competent state was neither affected by the dosage of D39-*ssbB-luc* inoculum, the exogenous provision of CSP, nor the bacterial burden in the lungs (26). Furthermore, the bacterial burden in the infected lungs decreased slightly between 0 and 24 hpi and only increased at the endpoints when mice entered the moribund state at which the infection had already spread systemically (26). The 20- to 24-hour duration required for the establishment of the competent state raises the possibility of an adaptive process in pneumococcus that is a prerequisite for entering the competent state during lung infection.

To unravel the underlying adaptive mechanisms driving the natural competence development in the lung, we conducted a time-resolved transcriptomic analysis guided by the spatiotemporal live IVIS imaging of competence induction in D39-*ssbB-luc* during pneumonia-derived sepsis in mice. The lung-residing pneumococcal transcriptome was examined using RNA sequencing (RNA-seq) at 0, 12, 24, and, at the moribund state, >40 hpi. In particular, different classes of genes were interrogated in the context of host adaptation that may provide insights on the mechanism of pneumococcal competence induction *in vivo*. In particular, we report that the *pht* genes of pneumococcus are required for the adaptive process prior to *in vivo* competence induction and that the Pht proteins may play an important regulatory role in the initiation of competence development with dependency on divalent cation availability not restricted to zinc.

## RESULTS AND DISCUSSION

### Murine model of pneumococcal lung infection and isolation of total RNA

**Fig 1:**
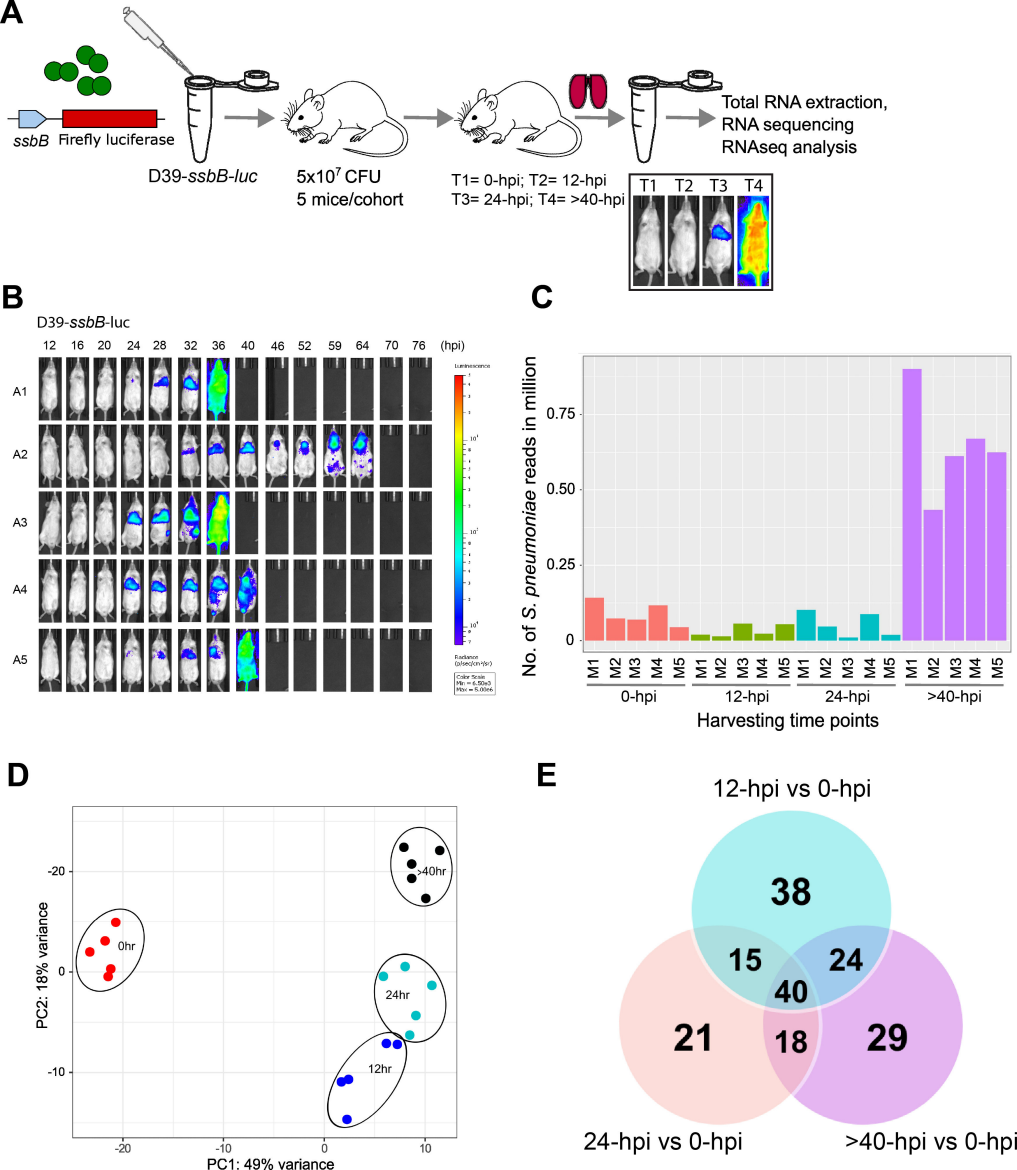
RNA-seq captures full repertoire of pneumococcal transcripts within murine lungs. (**A**) Schematic workflow of time-resolved RNA-seq comprising of T1 = 0 hpi, T2 = 12 hpi, T3 = 24 hpi, and T4 = >40 hpi in CD-1 mouse lungs (*n* = 5) intranasally infected with 5 × 10^7^ CFU of the D39-*ssbB-luc*. Before harvesting lungs for total RNA isolation, each cohort was imaged with IVIS SpectrumCT to examine spatiotemporal induction of the competent state. (**B**) Representative images captured by the IVIS SpectrumCT on the competence induction in mice infected by D39-*ssbB-luc*. (**C**) RNA read counts of D39-*ssbB-luc* across four time points. (**D**) PCA plot displays unique variance across the pneumococcal data sets demonstrating disease progression-specific clustering of the top 500 most variable pneumococcal genes. (**E**) Venn diagram displaying the number of genes expressed across the time points, organized into unique expressions and overlapping expressions.

Mouse infections, IVIS imaging, and total RNA isolation were schematically represented in Fig. 1A. Live IVIS imaging confirmed our previous finding (26) that none of the T1 (0 hpi) and T2 (12 hpi) groups entered the competent state, as indicated by the lack of bioluminescence signal. The T3 (24 hpi) cohorts displayed competence signals that were restricted to the lungs. Beyond T3, D39-*ssbB-luc* began to breach the alveolar-capillary barrier and spread systemically. The sepsis development progressed with a different speed in each animal (26), and lungs in the T4 (>40 hpi) group were harvested based on the moribund state and systemic bioluminescence signal outputs after 40 hpi as guided by the IVIS imaging (Fig. 1B).

For the purpose of transcriptome analysis, the recovered mouse lungs infected with pneumococcus were promptly subjected to total RNA isolation to quantify both host and the pneumococcal transcripts, allowing genome-wide quantitative snapshots of transcriptomes between host and pathogen. The total RNA-based transcriptomes have been reported to have fewer artifacts in expression levels that may arise from degraded RNA (33). Raw reads were trimmed and aligned to murine and pneumococcal reference genomes, and reads were counted separately as murine or pneumococcal. The analysis of murine transcriptome is currently in progress and will be published elsewhere. Bacterial reads were mapped to the reference genome (NC_008533.2) of pneumococcus strain D39. Lung samples recovered from T1, T2, and T3 groups yielded <0.14 million pneumococcal reads while lung samples from T4 group yielded higher between 0.43 and 0.9 million reads (Fig. 1C). Despite the comparably lower read counts in the T1, T2, and T3 groups, 100,000 reads are sufficient for identifying genes expressed at the levels of 10 reads per kilobase of transcript per million mapped reads. Furthermore, an even lower number of reads was sufficient to identify the majority of differentially expressed genes in the animal model of bacterial infection (34). The significantly elevated pneumococcal read counts at T4 are likely due to higher bacteria load and increased gene transcription as they have fully adapted to the host environment and weakened immune response in moribund mice. The read counts of the murine transcripts were consistent across all time points in excess of 10 million, eliminating the possibility of an anomaly due to sample handling. The transcription levels of each time point were normalized to the bacterial load to represent accurate fold-change in gene expression.

### Transcriptomic analyses indicate the importance of adaptation to the lung environment for triggering competence induction

Variations in the pneumococcal gene expression during lung infection were analyzed by principal component analysis (PCA) (Fig. 1D). The 500 most variable genes were selected in each lung sample, showing unique clustering in variance with the significance. T1 cluster demonstrated <49% variance from the rest of the groups, while T2, T3, and T4 showed <18% variance (Fig. 1D). The T1 cluster represents a baseline state of gene expression of <10 minutes after lung challenge from an active growth *in vitro* nutrient-rich culture. The high level of variance in T1 when compared to T2, T3, and T4 clusters indicates a large shift in gene expression profile from T1 (basal expression) to 12, 24, and >40 hpi, highlighting the adjustment in pneumococcal gene expression in the lung environment. PCA indicated two lung samples from the T2 time point (T2m3 and T2m5) clustering closer to the T3 cluster, while the remaining three T2 samples were adequately isolated. When considered together with the IVIS data showing the onset of *in vivo* competent state occurring at approximately 24 hpi, it suggests that the temporal window between T2 and T3 is the critical transition period in which the adaptive state switches to the naturally developed a competent state (Fig. 1B and D). The number of differentially expressed genes at T2 (12 hpi), T3 (24 hpi), and T4 (>40 hpi) ranged between 94 and 171, and many of these genes were common at all three time points (Fig. 1E; Fig. S1). As shown in the Venn diagram, when the gene expression patterns were compared to T1, 38, 21, and 29 upregulated genes were unique at T2, T3, and T4, respectively (Fig. 1E). Additionally, 15, 24, and 18 upregulated genes were shared between T2–T3, T2–T4, and T3–T4 time points, respectively. There were also 40 genes upregulated at all time points (Fig. 1E). Similarly, 20, 36, and 62 downregulated genes were unique at T2, T3, and T4, respectively. Additionally, 17, 2, and 31 downregulated genes were shared between T2–T3, T2–T4, and T3–T4, respectively. Finally, a total of 76 genes were downregulated at all time points (Fig. S1). The complete gene expression profile with log_2_ fold changes and *P*_adj_ values of pneumococcal genes across all time points is available in File S2 and can be downloaded as an Excel file.

**Fig 2:**
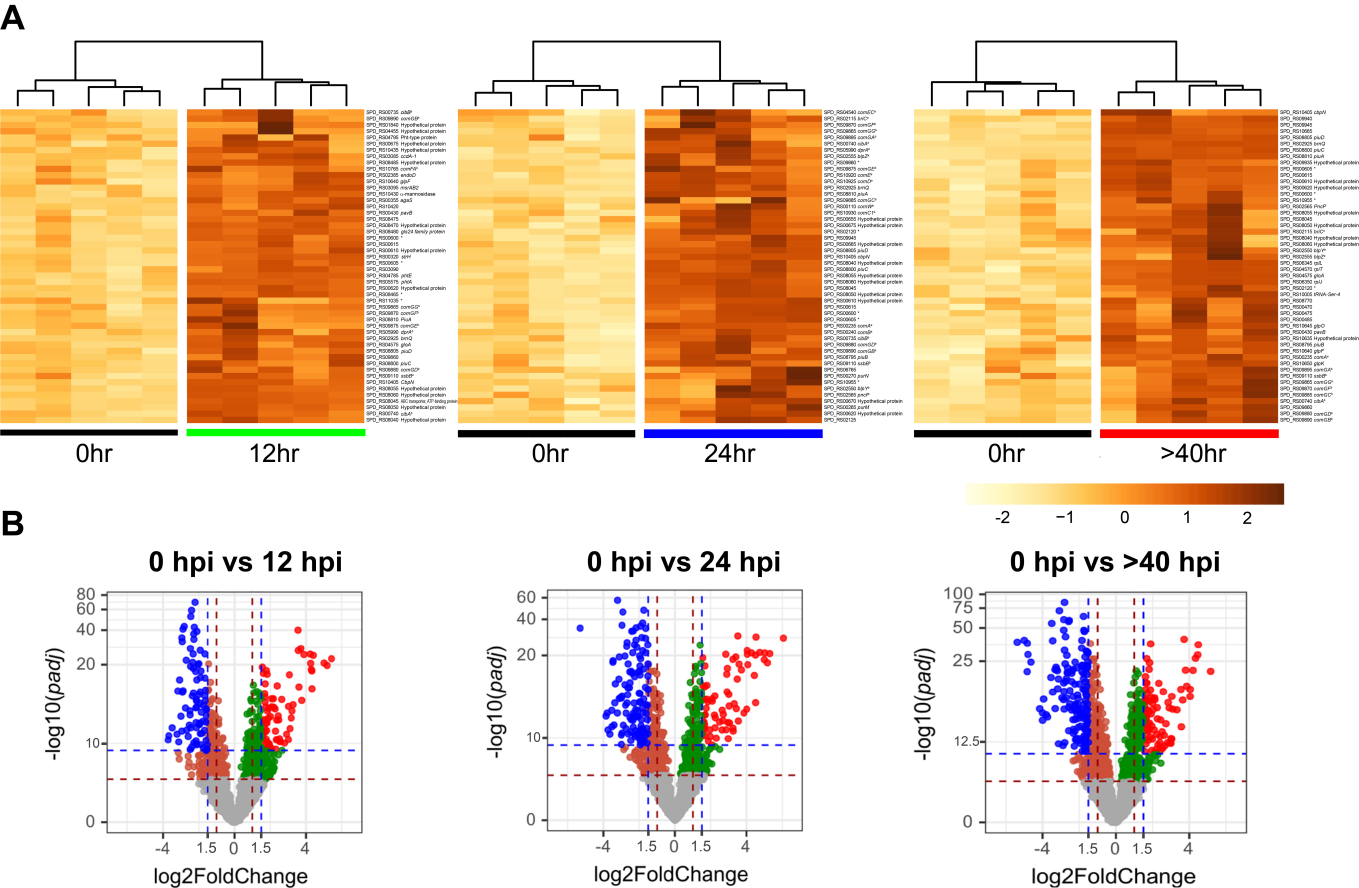
Heatmaps of differentially expressed pneumococcal genes from infected mouse lungs. (**A**) Each heatmap showcases up to 50 genes above the *P*_adj_ cutoff value that have undergone upregulation between the indicated time points based on the fold change. Scale bar (bottom right) indicates the gene expression level based on the log_2_ fold-change value between each time points. The superscripts indicate early, late, or delayed competence-specific genes (a: early, b: late, and c: delayed). The asterisk (*) indicates the unannotated locus tags. Blank annotations are shown in Table S3. All of the genes represented in the heatmaps are listed in Tables S5 to S7. (**B**) Volcano plots showing differentially expressed pneumococcal genes above the cutoff value set by the log_2_ fold change (*x*-axis) and the log_10_ adjusted *P* value (*y*-axis), indicated by color codes where blue and red dots represent downregulated and upregulated genes, respectively.

We generated a heatmap of the top 50 differentially expressed genes (log_2_ fold change > 1.5) for each time point (Fig. 2). Interestingly, the top 50 upregulated genes at T2 included some of the signature competence-specific genes such as *cibAB*, *dprA*, *ssbB*, *comGB*, *comGD*, *comGE*, *comGF*, *comGG*, and *comFA* (Fig. 2A; Tables S3 and S5 to S7). Our results indicate that, by at least T2, many “late competence genes” classified under *in vitro* competent state (16) are strongly upregulated during lung infection. Expression of these “late” competence genes as revealed by RNA-seq as early as T2 demonstrates the close association of pneumococcal competence regulon to host adaptation in the infected lungs. The failure to detect the competence development at protein level by IVIS prior to T3 may be due to the lower sensitivity of IVIS in detecting fewer low number of competent state pneumococcal cells residing in lung microcompartments (Fig. 1B). The temporal differences between transcription (RNA-seq) of competence genes and the accumulation of translated SsbB-luc to a detectable amount by IVIS might also contribute to the discrepancy. Another determining factor might be the accessibility to the d-luciferin substrate in *vivo* needed for the IVIS. It is likely that there was more signal once the pneumococci entered circulation due to the luciferin being more available when compared to pneumococci residing in lung microcompartments. The heatmap of the top 50 downregulated genes is shown in Fig. S2 (also see Table S3).

### Functional annotations of the differentially expressed genes

**Fig 3:**
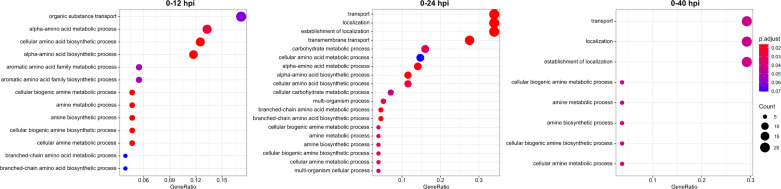
Functional annotation of differentially expressed pneumococcal genes in mouse lung across infection timespan when compared to initial basal expression. Each point symbol represents the ratio of genes pertaining to the corresponding gene ontology term to total number of upregulated genes. The color of symbols reflects the adjusted significance (*P*_adj_) by the Benjamini-Hochberg method, as illustrated in the scale. The size of the symbol reflects the number of genes represented, as indicated by the count scale. Detailed list of genes represented by the symbols is provided in Table S4.

Volcano plots were generated to show differentially expressed genes above the stringency cut-offs presented by log_2_ fold change and log_10_(*P*_adj_) values (Fig. 2B). The functions of the differentially expressed genes at each time point were annotated by the gene ontology term enrichment analysis (Fig. 3; Table S4). The enrichment analysis showed that upregulated genes include those that participate in the cellular processes involving organic substance transport, amine biosynthesis and metabolism, alpha-amino acid biosynthesis and metabolism, aromatic amino acid biosynthesis and metabolism, and branched-chain amino acid biosynthesis and metabolism. The dominant expression of genes involved in the amino acid biosynthesis and metabolism indicates that these biological processes are prioritized by lung-residing pneumococci at T2, underscoring the change in metabolic and biosynthetic processes after introduction into a hostile host environment (Fig. 3; Table S4). By T3, pneumococcal genes specialized in cellular processes such as transport, localization, establishment of localization, and transmembrane transport were dominantly expressed, followed by carbohydrate metabolism, amino acid biosynthesis, and metabolism (Fig. 3; Table S4). By T4, the expression of pneumococcal genes specialized in cellular processes such as transport, localization, establishment of localization, and transmembrane transport continued to dominate. Interestingly, cellular amine biosynthesis and metabolism are retained, suggesting their importance during systemic sepsis (Fig. 3; Table S4).

Significantly, as shown in the heatmap (Fig. 2), 21 of the top 50 upregulated genes at T3 are competence-specific genes, including *comABCDE*, *comW*, *blpYZ*, *cibAB*, *dprA*, and *ssbB* and the *comGB*, *comGD*, *comGE*, *comGF*, *comGG*, and *comFA* genes encoding for competence pili, suggesting the importance of various components within the competence system for a successful transition from T2 (12 hpi) to T3 (24 hpi). Together, *comA* and *comB* encode an ABC transporter involved in the export of CSP whereas the genes within the *comG* operon encode a pilus necessary for DNA uptake during genetic transformation. ComW participates in the activation and stabilization of the ComX, an alternative sigma factor that regulates the expression of ~80 “late” competence genes. Although not among the top 50 most upregulated genes, the expression of *comDE* genes (see Fig. 5B below), which encode the histidine kinase sensor (ComD) and the transcription factor (ComE), two-component regulatory systems that positively regulate the expression of “early” competence genes, showed sharp upregulation at T3. Strong upregulation of *comW* expression during the transition between T2 (12 hpi) and T3 (24 hpi) ensures the maximal activation and stabilization of the ComX required for the transcription of the “late” competence genes, among which are the competent state-specific virulence and allolytic factors that release pneumolysin and breach the alveolar-capillary barrier, ensuring a successful infection during pneumonia-derived sepsis (24, 26). Collectively, our current RNA-seq profiling data and our previous IVIS finding (26) suggest that the 12-hour window between T2 and T3 is a crucial period for pneumococcal adaptation during which the competence-specific genes undergo major upregulation, and the genes involved in adaptation may have implications in the development of virulence and pneumonia-derived sepsis *in vivo*.

### Validation of pneumococcal transcriptomics

**Fig 4:**
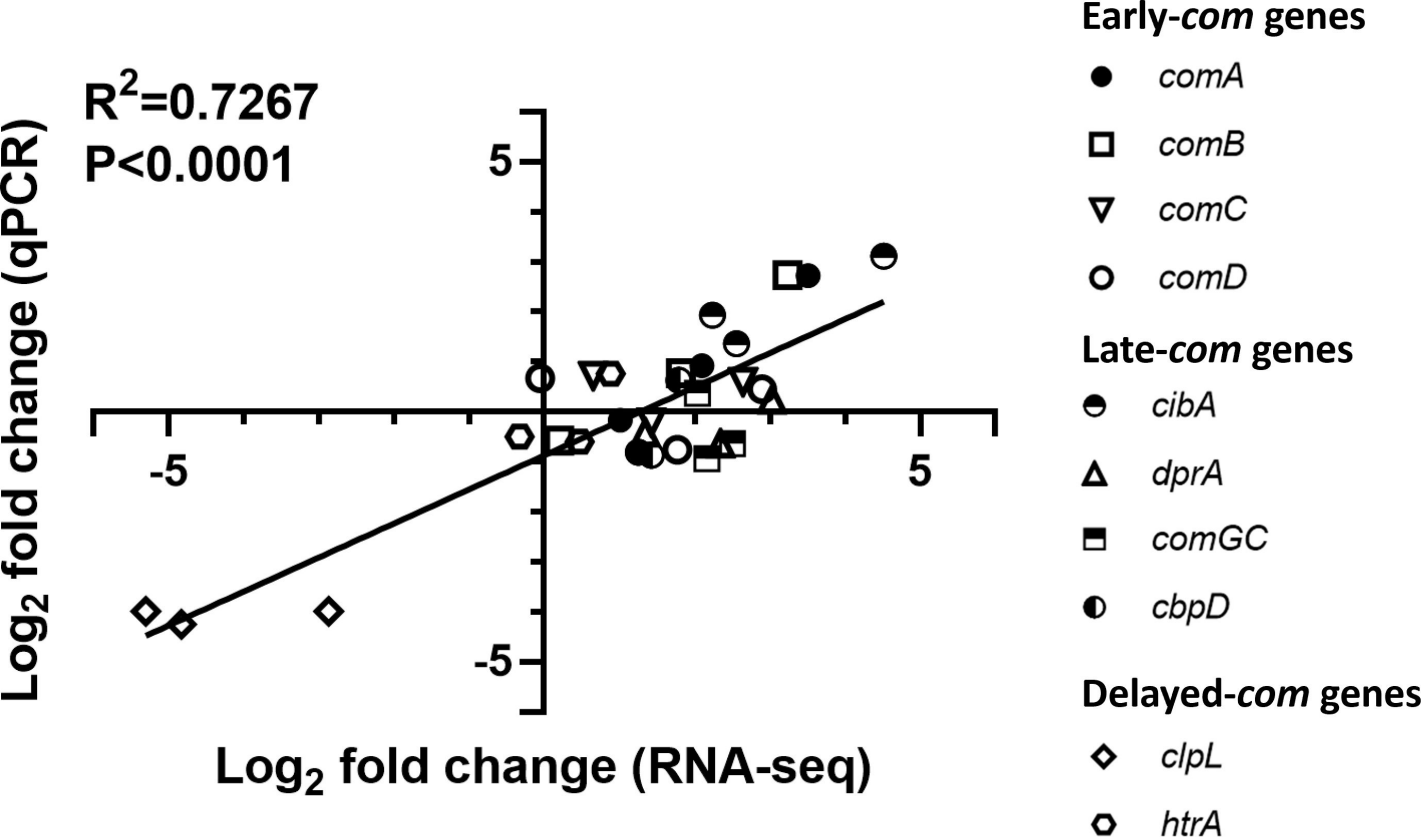
qRT-PCR validation of the expression of representative competence-specific genes revealed by RNA-seq. The same RNA samples isolated from the D39-*ssbB-luc*-infected lungs were used for qRT-PCR. Log_2_ fold-change values reflecting the differential gene expressions as validated by qRT-PCR. Ten competence-specific genes were analyzed, resulting in 30 plotted samples. A high degree of correlation was observed with the associated *P* value of <0.0001 (*R*^2^ = 0.727, Pearson). *X*-axis: log_2_ fold change (RNA-seq). *Y*-axis: log_2_ fold change (qPCR).

To validate the RNA-seq results, quantitative real-time quantitative reverse transcription PCR (qRT-PCR) was performed in triplicate using the total RNA samples isolated from the lungs used for the RNA-seq. The log_2_ fold-change values of 10 pneumococcal “early” (*comA*, *comB*, *comC*, and *comD*), “late” (*cibA*, *dprA*, *comGC*, and *cbpD*), and “delayed” (*clpL* and *htrA*) competence genes over four time points (T2–T1, T3–T1, and T4–T1) were selected for validation, using the primers listed in Table S2. The resulting total of 30 log_2_ fold-change values from the qRT-PCR analysis were compared to that of RNA-seq, which resulted in a high degree of correlation with the *P* value of <0.0001 (*R*^2^ = 0.7267, Pearson) (Fig. 4).

### Expression profiles of carbohydrate metabolism genes

**Fig 5:**
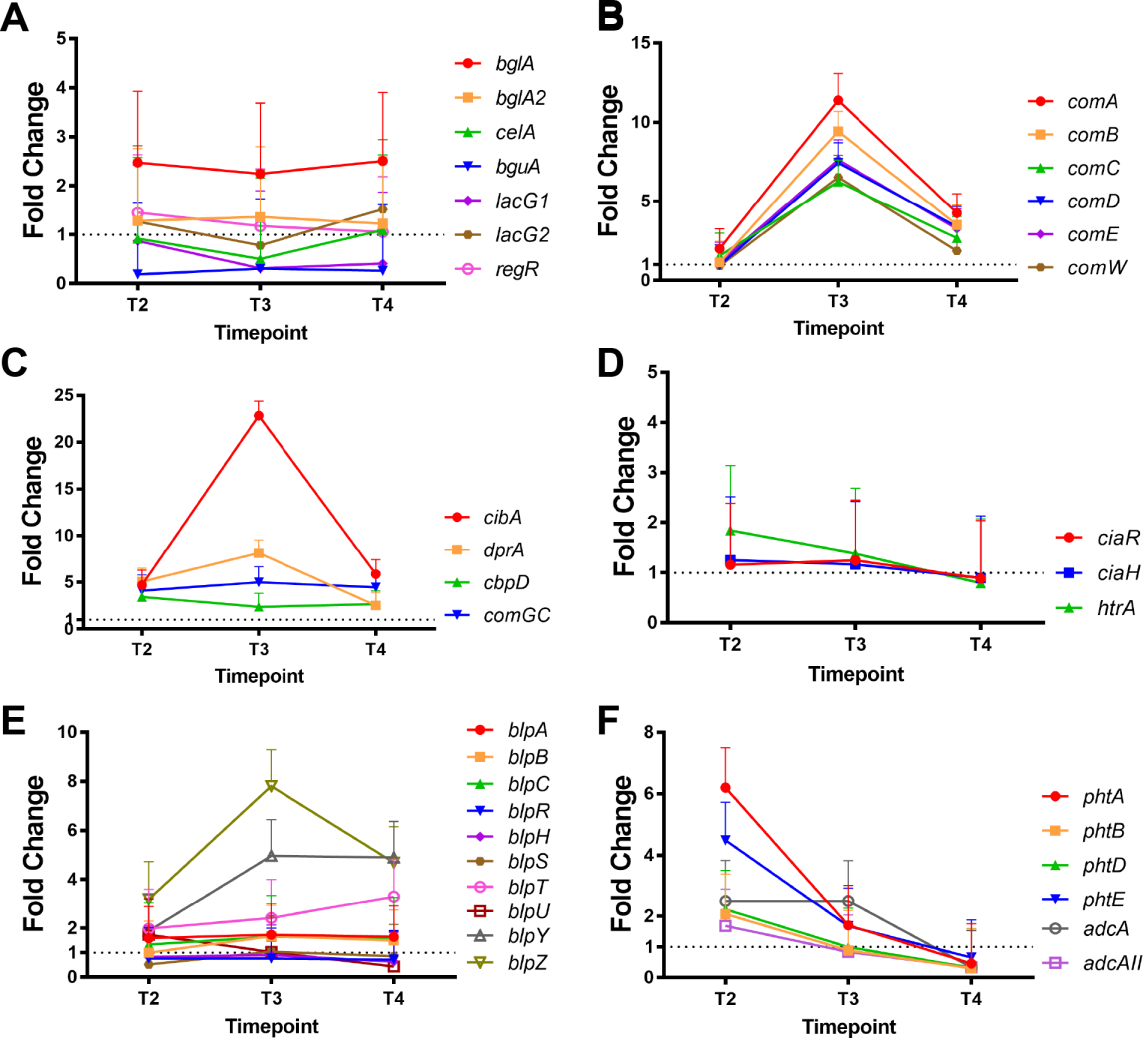
Changes in expression of selected genes across timespan in infected lungs shown in linear scale. (**A**) Seven representative pneumococcal genes encoding 6-phospho-β-glucosidases (*bglA*, *bglA2*, *celA*, and *bguA*) and 6-phospho-galactosidases (*lacG1* and *lacG2*). RegR regulates lacG1 and LacG2. (**B–D**) Early, late, and delayed competence-specific signature genes. (**E**) Competence-regulated genes of the *blp* locus encoding bacteriocin-inducing peptides and immunity proteins of the BIR. (**F**) The *phtABDE and adcA* and *adcAII* genes involved in zinc acquisition. The numerical values of fold change and corresponding standard error values are provided in Table S8.

The hydrolysis activity by phospho-β-glucosidases (Enzyme Commission number 3.2.1.86) and phospho-β-galactosidases (Enzyme Commission number 3.2.1.85) is central to bacteria obtaining glucose and glucose 6-phosphate for glycolysis and energy generation within animal hosts (35, 36). Of the six genes annotated as the encoding for the 6-phospho-β-glucosidases (*bglA*, *bglA2*, *celA*, and *bguA*) or 6-phospho-β-galactosidases (*lacG1* and *lacG2*), *bglA* [SPD_RS01345, same as *bglA3* “SPD_0247” as described by Terra et al. (35)] retained markedly high levels of expression throughout the infection, showing 2.48-, 2.25-, and 2.51-fold increase at T2, T3, and T4, respectively (Fig. 5A). The expression of other genes encoding phospho-β-glucosidases and phospho-β-galactosidases was not significantly altered throughout the infection (Fig. 5A; Table S8). BglA deficiency leads to reduced bacterial burden and attenuated ability for pneumococcus to cause mouse mortality during lung infection (35). Specifically, the lung burdens of the *bglA* mutant at 12 hpi were not significantly different from those of the isogenic wild-type D39 but dropped precipitously to ≥log_10_ 4.34 ± 0.3 by 24 and 48 hpi (35), suggesting the importance of β-glucoside metabolism in bacterial survival and virulence in mouse lungs. Similarly, BglA had been shown to be important for virulence, adherence, and biofilm formation in *Streptococcus gordonii* (37). When aligned with our transcriptomic data, these findings highlight the importance of *bglA* expression prior to 24 hpi for host adaptation. The pneumococcal competence system has been shown to be tightly associated with virulence (18, 21, 23–26, 38, 39), adherence to host cells (40, 41), and biofilm formation (20, 40, 42). Importantly, *regR* (SPD_RS01645), a global LacI/GalR transcriptional regulator (Fig. 5A), has previously been shown to play a role in adaptive response in *S. pneumoniae* by regulating competence, adherence, and expression of virulence factors such as hyaluronidase (43). The peak expression level of *regR* was at T2 (1.46-fold) followed by a gradual decline, indicated by 1.18-fold (T3) and 1.06-fold (T4) expression in respect to T1 (Fig. 5A).

### Differential expression of competence-specific genes

As shown in Fig. 2 when compared against T1, 20%, 42%, and 24% of the top 50 most upregulated pneumococcal genes in infected mouse lungs at T2, T3, and T4 are the “late” competence genes (Fig. 2; Tables S3 and S5 to S7). Our transcriptomic data show that the concerted massive expression of “late” genes was initiated by the expressions of “early” competence gene *comCDE* operon, which drove a positive feedback loop for complete competence induction, peaking at T3 (Fig. 5B). Interestingly, when compared to T1 (0 hpi), the transcript levels of *comC* at T2 (12 hpi) were moderately increased by 1.57-fold, but *comD* and *comE* were stable at 0.96-fold and 1.14-fold, respectively. Nevertheless, these amounts of *comCDE* gene expression appear to be adequate to encode proteins necessary to drive a significant increase in the expression of “late” competence genes including *cibA*, *dprA*, *cbpD*, and *comGC* (Fig. 5C). Interestingly, the expression of *ciaRH* genes, which encode the two-component regulatory systems that negatively regulate competence, was maintained at low levels and minimally changed when compared to that of T1, with their linear fold change values of 1.16- and 1.26-fold change at T2 and 1.25- and 1.17-fold change at T3 (24 hpi) followed by downregulation below T1 levels at T4 (Fig. 5D). For *htrA*, a 1.84-fold increase was observed at T2 followed by gradual decline until T4 (Fig. 5D). Under *in vitro* conditions, CiaRH negatively regulates *comCDE* transcription, and HtrA degrades CSP pheromone (44, 45). This suggests that controlled upregulation of *ciaRH* and particularly *htrA* during the early stage of infection might be important for optimal induction of a competent state during pneumonia-derived sepsis. Our results indicated that *ciaRH* and *htrA* were not expressed at T4 (>40 hpi) despite the highly active competent state, suggesting that CiaRH may not be involved in the competence shutoff *in vivo* but rather in controlling the onset of the competent state during the early stages of lung infection. For example, maximum expression levels for *comCDE* were achieved at T3 with 6.25- to 7.63-fold increase, and the upregulation in these genes was maintained at T4 above 2.69-fold (Fig. 5B). This upregulation in *comCDE* gene expression is consistent with our previous observation that pneumococcus is able to maintain a persistent and prolonged competent state after the breach of alveolar-capillary barrier until animals are moribund (Fig. 1B) (26), which is a unique feature of pneumococcal pneumonia-derived sepsis that distinguishes from the transient short burst of *comCDE* observed *in vitro* (16). The numerical values of fold change and corresponding standard error values are provided in Table S8.

Previously, a dual RNA-seq in the zebrafish model of early meningitis infection has shown competence induction as early as 2 hours after injection of pneumococcus into the hindbrain ventricle of the fish larvae (15). Importantly, the authors have shown that competence induction occurred in non-synchronized manner among a subset of infecting pneumococci, supporting the stochastic induction of the competent state over concerted and prolonged induction of the competent state. In our pneumonia-derived sepsis model, we are unable to image competence induction at a single pneumococcal cell level. However, our current study shows that the competence genes are strongly upregulated as early as T2 and continuously maintained at the population level within lung microcompartments until after the breach of alveolar-capillary barrier resulting in a systemic competence at which point, animals became moribund (26). We hypothesize that the pneumococcal population within the host tissue microcompartments initiates the competence development during the early stage of infection (e.g., T2) as an adaptation response to the lung environment, but is continuously needed as pneumococci spread systemically, thus maintaining the competent state throughout the entire infection course.

### Competence-regulated bacteriocins (pneumocins) and immunity genes

Bacteriocin-inducing peptide has been implicated in pneumococcal competence induction. The *blpC* gene encodes double-glycine peptide BlpC, a quorum sensing autoinducer that activates the BlpRH two-component signaling system to upregulate four to six operons in *blp* loci (46), including genes found in bacteriocin-immunity region (BIR) (46–49). Importantly, competence induction by CSP also activates, but not a prerequisite to, the transcription of *blp* genes in BIR under *in vitro* condition through the binding of ComE onto the promoters of genes with the *blp* loci, thus augmenting the fratricide activity during competent state (46, 49) as well as during antibiotic exposure (50). We show that the expression of *blpC* and the ABC transporter for the *blp* locus (*blpAB*) were increased throughout the infection (Fig. 5E). In contrast, the expression of *blpR*, the transcriptional regulator of the Blp regulon, remained slightly downregulated at 0.76- (T2), 0.76- (T3), and 0.72-fold (T4) relative to T1 (0 hpi) (Fig. 5E). The bacteriocin immunity proteins *blpY* and *blpZ* underwent marked upregulations, both peaking at the T3 (24 hpi) with 4.97- and 7.81-fold increase, respectively (Fig. 5E). Of the three bacteriocin genes *blpS*, *blpT*, and *blpU*, only the expression of *blpT* showed gradual increase by the order of 1.99-, 2.42-, and 3.30-fold, in T2 (12 hpi), T3 (24 hpi), and T4 (>40 hpi), respectively. These results suggest that the expression of the genes encoding bacteriocins and their cognate immunity proteins (*blpT*, *blpY*, and *blpZ*) may contribute to pneumococcal adaptation and survival. However, the fact that the expression of the *blpRH* remained downregulated suggests that it is the ComDE that exerts transcriptional control over the *blp* operons throughout the entire pneumonia-derived sepsis infection.

### Pneumococcal histidine triad (*pht*) proteins are divalent metal-dependent regulators of competence induction in early adaptation to the lung environment

The high levels of upregulation of the “late” competence genes at T2 suggest that the invading pneumococci in the lungs have entered the competent state by 12 hpi (Fig. 2). Thus, we focused on selected genes displaying exclusively higher expression levels at T2 in order to interrogate putative bacterial factors that may contribute to the adaptation process prior to full induction of pneumococcal competence *in vivo*. Among others, one family of four genes encodes the pneumococcal histidine triad proteins, *phtA* (SPD_RS05575), *phtB* (SPD_RS05570), *phtD* (SPD_RS04780), and *phtE* (SPD_RS04785) (Fig. 5F). These genes are regulated by the *adc* locus, which is known to mediate the Zn^2+^ and Mn^2+^ uptake (41, 51–53). Pht proteins localize to the cell surface and are detectable in the supernatant as soluble proteins (54). They are better known as promising vaccine candidates, triggering protective humoral immunity in the host (52, 54–56). Immunization with Pht proteins protects mice against nasopharyngeal colonization, complement deposition, and adherence to host cell surfaces by pneumococcus (55–59). Importantly, Pht proteins have been reported to participate in the maintenance of zinc homeostasis in pneumococcus, where activation of transcriptional repressor AdcR under high concentration of zinc results in the inhibition of transcription of *pht* genes (55, 56). The Zn^2+^ acquisition in *S. pneumoniae* involves the ATP-binding cassette transporter AdcBC and two zinc-binding proteins, AdcA and AdcAII, belonging to A-1 cluster family of substrate-binding proteins (SBPs). The AdcA and AdcAII are functionally redundant in Zn^2+^ acquisition. AdcA has the capability of independently recruiting Zn^2+^, while AdcAII requires Pht proteins (60). However, the biological functions of Pht proteins in metal homeostasis and their link to competence induction have not been previously established.

**Fig 6:**
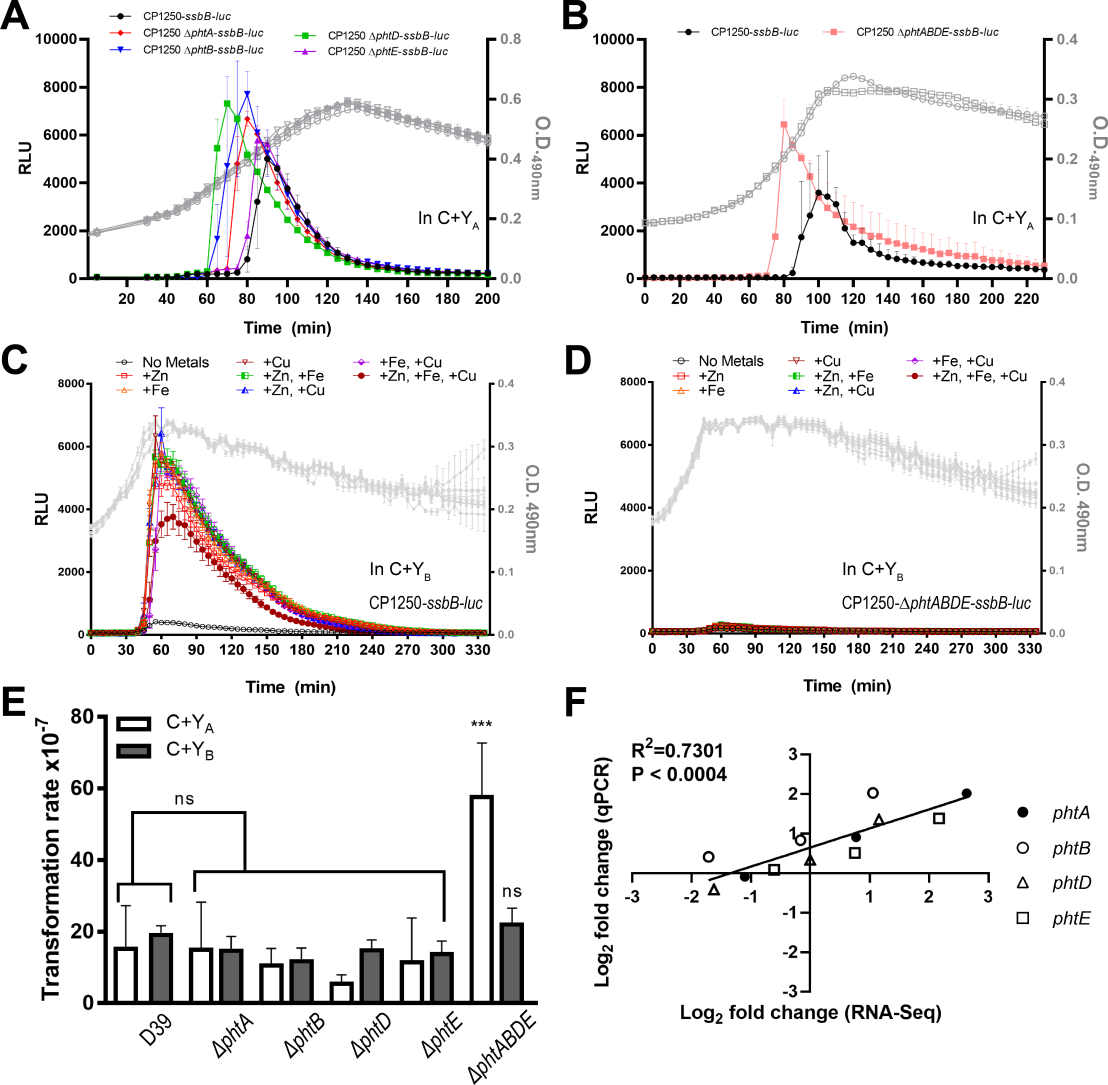
Loss of *pht* genes leads to metal-dependent dysregulated competence phenotypes *in vitro*. The firefly *luc* reporter gene was genetically fused to competence-specific *ssbB* in a hypercompetent pneumococcal strain CP1250 to monitor spontaneous competence induction in *pht* mutants. (**A**) In a metal-rich (+Cu^2+^, +Fe^2+^, and +Zn^2+^) C + Y_A_ (pH = 7.8), *∆phtA*, *∆phtB*, *∆phtD*, and *∆phtE* show varying degrees of expedited onset of the competent state as well as higher levels of competence. (**B**) In C + Y_A_ (pH = 7.8), *∆phtABDE* enters competence state earlier with higher degree of competence. (**C**) In metal-deficient (−Cu^2+^, −Fe^2+^, and −Zn^2+^) C + Y_B_, CP1250-*ssbB-luc* fails to enter competent state naturally unless provided with 432 µg L^−1^ of CuSO_4_, FeSO_4_•7H_2_O, ZnSO_4_•7H_2_O, or any combination of the divalent metals. (**D**) In C + Y_B_, CP1250-Δ*phtABDE-ssbB-luc* fails to enter competent state naturally even with supplementation of 432 µg L^−1^ of CuSO_4_, FeSO_4_•7H_2_O, ZnSO_4_•7H_2_O, or any combinations of the divalent metals. Typical results from one of the three independent experiments are shown. (**E**) In metal-sufficient C + Y_A_, Δ*phtABDE* mutant derived from the pneumococcal strain D39 displays increased rate of genetic transformation frequency. *P* = 0.0001 by one-way analysis of variance test. (**F**) The qRT-PCR validation of the RNA-seq expression of the *pht* genes from the infected mouse lungs with the *P* value of 0.0004 (*R*^2^ = 0.7301, Pearson). *X*-axis: log_2_ fold change (RNA-seq). *Y*-axis: log_2_ fold change (qPCR).

The expression of *phtA* and *phtE* was increased by 6.20- and 4.49-fold, respectively, two of the top 50 upregulated genes, while *phtB* and *phtD* showed 2.08- and 2.23-fold increase at T2 relative to T1, respectively (Fig. 5F). Interestingly, the expression of all *pht* genes underwent steep decline after T2 (*phtA*, T2: 6.2048; T3: 1.7068; and T4: 0.4650; *phtB*, T2: 2.0777; T3: 0.8910; and T4: 0.3048; *phtD*, T2: 2.2309; T3: 0.9990; and T4: 0.3241; and *phtE*, T2: 4.4916; T3: 1.6845; and T4: 0.6564) (Fig. 5F). To further characterize the role of *pht* genes in the competence processes, we used an *in vitro* reporter assay and compared the induction of natural competence in the hypercompetent CP1250-*ssbB-luc* parental strain versus mutants deficient in individual *pht* genes and *∆phtABDE*. Spontaneous competence induction was monitored using two distinct formulations of C + Y medium that have been previously described in a competence-permissive pH 7.8: C + Y_A_ (61) and C + Y_B_ (62) (detailed compositions of C + Y_A_ and C + Y_B_ medium are listed in Table S9). In terms of divalent metal ion contents, both recipes contain calcium chloride (CaCl_2_), magnesium chloride hexahydrate (MgCl_2_•6H_2_O), and manganese sulfate tetrahydrate (MnSO_4_•4H_2_O), but C + Y_A_ has more CaCl_2_ and MnSO_4_•4H_2_O than C + Y_B_. Furthermore, C + Y_A_ contains cupric sulfate (CuSO_4_), ferrous sulfate heptahydrate (FeSO_4_•7H_2_O), and zinc sulfate heptahydrate (ZnSO_4_•7H_2_O), all of which are absent in the C + Y_B_ (Table S9). Strikingly, in the C + Y_A_ medium, all of the *pht* mutant strains elaborated higher competent state as illustrated by bigger peak value of *ssbB* expression compared to the CP1250-*ssbB-luc*, in the order of *∆phtB-ssbB-luc* (53.8%), *∆phtD-ssbB-luc* (45.9%), *∆phtA-ssbB-luc* (33.2%), and *∆phtE-ssbB-luc* (15.2%) (Fig. 6A). The *∆phtABDE-ssbB-luc* showed a 43.7% higher *ssbB* expression compared to CP1250-*ssbB-luc* (Fig. 6B). Furthermore, a temporal shift in the induction of competent state was observed. Detailed examination indicates that both *∆phtB*, *∆phtD*, and *∆phtABDE* have similar temporal onset of the competence induction, which is 10 minutes earlier than the parental strain CP1250. However, the amount of time for each of these strains to reach the peak competent state differs, with *∆phtB* 20 minutes, *∆phtD* 10 minutes, and *∆phtABDE* 20 minutes, earlier than the peak competent state achieved by CP1250 (Fig. 6A and B). Based on these observations, it is likely that PhtB plays a more dominant role in modulating competence induction and competent state in pneumococcus under metal-sufficient C + Y_A_-defined medium. Considering that the *in vitro* competent state in pneumococcus only lasts less than 40 minutes, the expedited onset of the competent state with varying degrees of hypercompetence in various *∆pht* mutants suggests a repressive role of Pht proteins in pneumococcal competence induction in the transition metal-rich C + Y_A_ medium. The earlier onset of the competent state in *∆pht* mutants did not lead to a quicker decline, suggesting that Pht proteins do not contribute to the competence shutoff (Fig. 6A and B).

In the C + Y_B_ medium, which lacks Zn^2+^, Fe^2+^, and Cu^2+^, both the parental strains CP1250*-ssbB-luc* and CP1250-*∆phtABDE-ssbB-luc* failed to enter a spontaneous competence state in a competence-permissive pH of 7.8 (Fig. 6C and D). However, in CP1250*-ssbB-luc*, the competence deficiency phenotype was rescued after the addition of Zn^2+^, Fe^2+^, or Cu^2+^ individually or in combinations (Fig. 6C). In CP1250-*∆phtABDE-ssbB-luc*, however, neither individual nor combination of Zn^2+^, Fe^2+^, and Cu^2+^ was able to trigger spontaneous competence induction (Fig. 6D). These results indicated that inability to acquire Zn^2+^, Fe^2+^, or Cu^2+^ through the PhtABDE-AdcAII system contributed attenuated competence induction in the deletion mutant CP1250-*∆phtABDE-ssbB-luc*, which losses the ability to uptake divalent cations. Furthermore, AdcA alone may not be sufficient to initiate the energy-expensive pneumococcal competence development. To authenticate the findings in Fig. 6C and D, the competence-derived transformation efficiency of *pht* mutants in both C + Y_A_ and C + Y_B_ media was assessed in wild-type pneumococcal strain D39 with the addition of CSP1 (Fig. 6E). In the transition metal-rich C + Y_A_, D39-*∆phtABDE* displayed higher transformation rate that was not observed in metal-deprived C + Y_B_ (Fig. 6E). It should be noted that the difference in compositions of C + Y_A_ and C + Y_B_ media is more than the contained metals (Zn^2+^, Fe^2+^, or Cu^2+^), and the two distinct C + Y_A/B_ media have different amino acid content (Table S9). Therefore, the competence induction in ∆*phtABDE* mutant may rely on the availability of various amino acids and not solely on the presence of divalent metals. However, the competence rescue observed in CP1250-*ssbB-luc* but not in CP1250-*∆phtABDE-ssbB-luc* by metal supplementations in the amino acid-rich C + Y_B_ suggests a close association between divalent metals acquisition and competence induction facilitated by the Pht proteins.

Finally, qRT-PCR validated the differential expression of *pht* genes from RNA-seq during pneumonia-derived sepsis and derived a high degree of correlation (*R*^2^ = 0.7301, Pearson) over four time points (T2–T1, T3–T1, and T4–T1) (Fig. 6F), using the primers listed in the Table S2. The *P* value associated to the Pearson’s coefficient was 0.0004.

### Pht proteins are required for optimal competence induction during pneumonia-derived sepsis in mice

**Fig 7:**
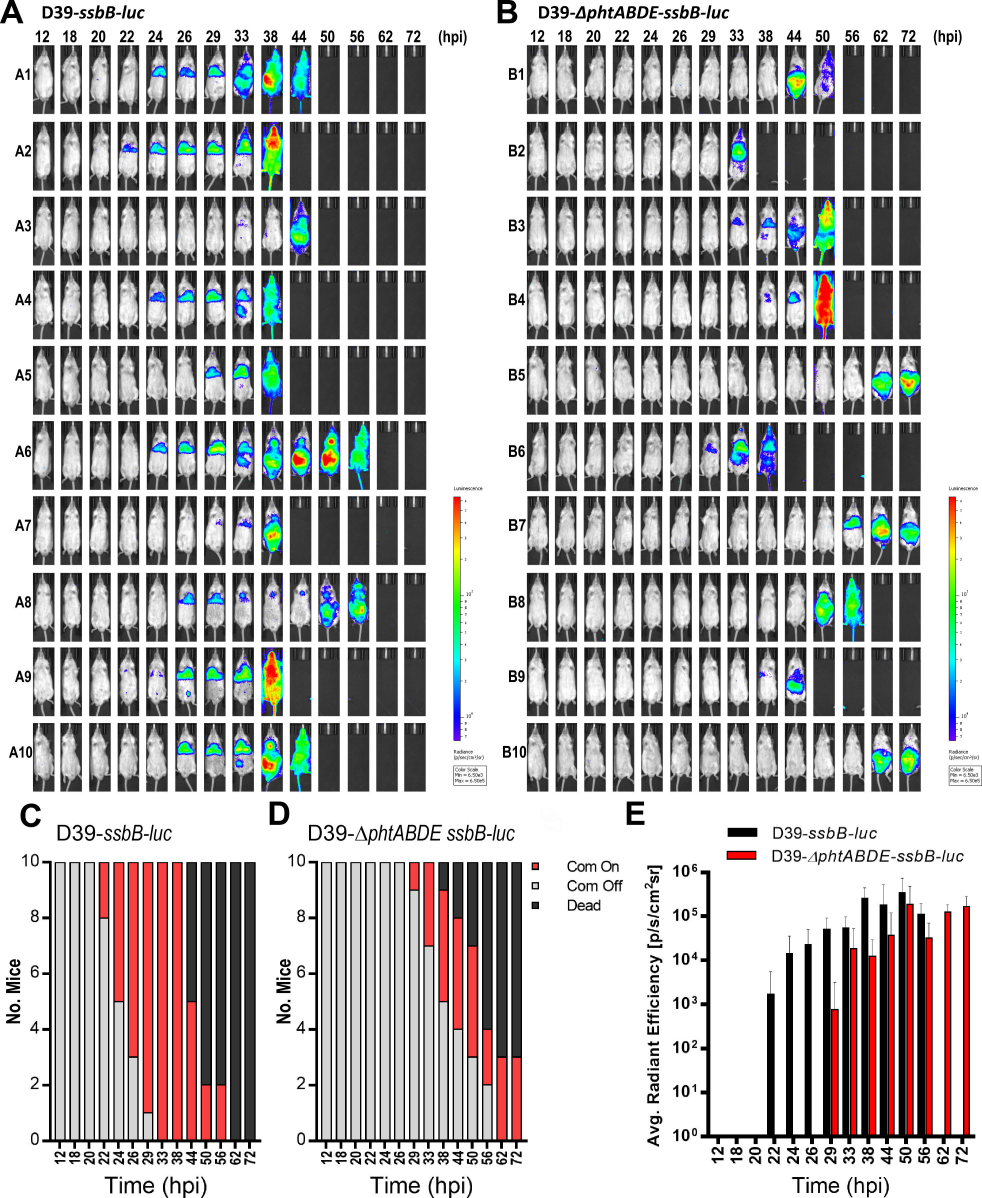
Pht proteins regulate entry into spontaneous competent state during pneumonia-derived sepsis. (**A and B**) CD-1 mice (7 weeks old, 10/group) were intranasally inoculated with 5 × 10^7^ CFU of D39-*ssbB-luc* (**A**) or D39-Δ*phtABDE-ssbB-luc* (**B**), and competence induction was monitored for 72 hours with IVIS imaging by detecting the bioluminescent signals. (**C and D**) Stacked bar graphs were constructed to indicate numbers of mice displaying either non-competent pneumococci (gray), competent pneumococci (red), or euthanized (black) status at each time point. (**E**) Average radiant efficiency of each group was quantified at each time point to determine the level of competence induction with threshold set to 20%.

Our *in vitro* data suggest that Pht proteins negatively regulate spontaneous competence induction in the C + Y_A_ medium containing Ca^2+^, Mg^2+^, Fe^2+^, Cu^2+^, Zn^2+^, and Mn^2+^ at competence permissive pH 7.8 (Fig. 6A and B). Conversely, in C + Y_B_ medium lacking Fe^2+^, Cu^2+^, and Zn^2+^, which does not support spontaneous induction of competence in CP1250-*ssbB-luc* even at pH = 7.8*,* Pht proteins positively regulate spontaneous competence induction by recruiting the divalent Fe^2+^, Cu^2+^, and Zn^2+^ metal ions (Fig. 6D and E). Next, we determined whether the Pht proteins played a role in competence induction during acute pneumonia-derived sepsis in mice (Fig. 7). We intranasally infected CD-1 mice (*n* = 10) with D39-*ssbB-luc* or D39-*∆phtABDE -ssbB-luc* reporter strains and tracked competence induction spatiotemporally with the IVIS imaging. Consistent with our previous finding (26), D39-*ssbB-luc* displayed competence induction as early as 22 hpi. By 26 hpi, pneumococci in 7/10 mice showed competence signals, and by 33 hpi, all 10 mice displayed competence induction by in the infected lung (Fig. 7A and C). Strikingly, D39-*∆phtABDE-ssbB-luc* displayed significantly delayed entry into the competent state, with the first mouse observed at 29 hpi (Fig. 7B and D). Additionally, D39-*∆phtABDE-ssbB-luc*-infected mice B5 and B10 did not develop a competent state until as late as 62 hpi (Fig. 7B), whereas mice B1, B7, and B9 developed competent state at 44, 56, and 38 hpi, respectively (Fig. 7B). However, region of interest (ROI) quantification with a 20% threshold indicated no difference in the intensity of competence induction between the parental strain and the *pht*-deficient mutant (Fig. 7E), suggesting that the defect was in the delayed entry into the competent state. However, once D39-*∆phtABDE-ssbB-luc* attains the competent state, the levels of induction are similar to the parental strain D39-*ssbB-luc*.

Collectively, our results indicate that Pht proteins play an important role in the adaptive process necessary for successful entry into a competent state following acute pneumoniae infection by mediating metal acquisition in lung compartments where the availability of transition metals is scarce in the lung.

### Summary

Most of the mechanisms involved in the competence regulon-mediated pneumococcal genetic transformation were derived from the *in vitro* studies. A few *in vivo* studies have now begun to analyze competence development during host infection (15, 20, 21, 23–26). Apart from regulating DNA uptake and genetic recombination, the sigma factor ComX also elevates the expression of competent state-specific *cibAB*, *cbpD*, and *lytA* genes that encode allolytic factors (24) responsible for the release of pore-forming pneumolysin that erodes the alveolar-capillary barrier, allowing subsequent systemic spread and the development of pneumonia-derived sepsis (26) and multi-organ dysfunction (63, 64). Most recently, we have shown that the serotype 2 strain D39, which typically does not enter the competent state naturally *in vitro*, could enter a prolonged competent state during acute pneumonia in mice (26). The competent state occurred approximately at 20–24 hpi, followed by the breach of alveolar-capillary barrier and systemic sepsis, where the competent state was maintained until mouse death. Interestingly, the provision of CSP or increasing the inoculum concentration of D39-*ssbB-luc* inoculum could not hasten the rigid 20–24 hours needed to initiate the competent state (26), suggesting the existence of stringent requirement(s) imposed by the lung environment that pneumococcus needs to overcome in order to naturally enter the competent state. Unfortunately, the mechanisms that regulate the adaptation in the lung that is permissive to competence development are unknown. To address the aforementioned knowledge gap, we performed a time-resolved RNA-seq analysis on the D39-*ssbB-luc* strain guided by the live IVIS imaging to gauge various gene requirements needed for pneumococci to enter the competent state during a mouse model of pneumonia-derived sepsis.

Nearly one-third of the pneumococcal genome encodes for proteins involved in sugar transport, degradation, and processing, especially via 14 operons of genes dedicated to sugar utilization during host infection (65). Among various classes of the upregulated genes potentially involved in adaptation, we found that elevated transcription of *bglA* and *bglA2* encodes membrane-localized 6-phospho-β-glucosidases that hydrolyze host-derived sugars, including beta-glucoside analogs such as the β-1,4-linked cellobiose 6-phosphate to yield glucose and glucose 6-phosphate that can be fed into glycolysis for ATP generation during pneumococcal fermentation (35). The expression of *bglA* and *bglA2* begins at 12 hpi or earlier after infection and was uniquely maintained throughout the entire cycle of infection, suggesting their crucial role in pneumococcal energy generation. When compared to *in vitro* growth with glucose, the expression of *blgA* was increased during lung infection, with its deficiency leading to pleiotropic defects including loss of virulence, impaired attachment, and attenuation in biofilm formation, all of which are closely linked to competence (35). Furthermore, higher expression of *regR* at T2 with diminishing levels at T3 and T4 suggests the likelihood of a positive relationship between sugar metabolism and pneumococcal competence system, as RegR has been shown to regulate competence via CiaRH signaling pathway (43).

Another group of upregulated genes at T2 potentially involved in the adaptation is the pneumocins encoded by the *blp* locus (66). In addition to regulation by BlpRH, the *blp* locus is also cross-regulated by the ComDE of the competence regulon (46, 49, 50). With the exception of *blpRH*, *blpS*, and *blpU*, the expression of remainder *blp* genes was upregulated between T2 and T4, even in the absence of *blpRH*, suggesting that ComCD exerts epistatic transcriptional control over the *blp* loci during lung infection and that the pneumocins may play a role in the pathogenesis of pneumonia-derived sepsis. Due to redundancy in the regulation of the *blp* loci by BlpRH and ComDE, placing the control of pneumocin expression under the competence regulon may be more energetically favorable. Alternatively, upregulation of bacteriocin immunity proteins may protect against self-predation from fratricide during *in vivo* competent state or promote niche adaptation in the lung microenvironment by killing off closely related competing bacterial species, ensuring successful infection.

Another group of T1 and T2 adaptation genes encodes the PhtABDE adhesins (67) that bind to epithelial cells, an initial and crucial step in the colonization and infection. PhtABDE proteins harbor multiple copies of histidine triad motifs (HxxHxH) that bind Zn^2+^ and Mn^2+^, and they are speculated to be involved in the acquisition of these metal ions and transferring the Zn^2+^ to AdcAII for uptake (68). *S. pneumoniae*, unique from most other species, possesses two SBPs dedicated to Zn^2+^ uptake. Despite the presence of the main Zn^2+^ uptake system (AdcA) (68), *∆phtABDE* mutant is unable to grow unless supplemented with either Zn^2+^ or Mn^2+^ (51). Because mammalian lungs are devoid of free Zn^2+^ and Mn^2+^, our RNA-seq and qRT-PCR data show that *phtABDE* genes are expressed primarily between T1 and T2 to acquire Zn^2+^ and Mn^2+^ for pneumococcal growth and are in line with PhtABDE serving as chelators of Zn^2+^ or Mn^2+^. Once sufficient levels of these metal ions are obtained, the expression of *phtABDE* genes is shut off by AdcR (51, 58). Interestingly, both Zn^2+^ and Mn^2+^ seem to be important for genetic transformation (41). In this study, we demonstrated that a certain amount of Zn^2+^, Fe^2+^, and/or Cu^2+^ is conducive for the CP1250 strain to enter spontaneous competence, which was not the case for *∆phtABDE* mutant (Fig. 6A and B). This suggests that Pht proteins, previously regarded as Zn^2+^ chelators, may also uptake other transition metals such as Fe^2+^ and Cu^2+^ for import via AdcAII and modulate competence induction. However, competence regulation by Pht proteins may be affected not solely by metal availability but also by the presence of various amino acids. We further demonstrated that the Pht proteins modulate competence induction during pneumonia-derived sepsis in mice (Fig. 7). Although the intensity of competence induction in *∆phtABDE* mutant was indistinguishable to that of the parental strain (shown by ROI quantifications), the loss of *phtABDE* genes significantly delays the entry to a competent state in the infected lungs.

Conditional fitness due to competence and culture condition has been suggested by Engelmoer et al. (69), who demonstrated that competence induction in a benign condition leads to reduction in fitness gain compared to non-competent pneumococci. In our experiment, *pht* mutant strains displayed an exaggerated competent state at a lower cell density in a metal-sufficient environment (C + Y_A_), which potentially may reduce long-term fitness from unnecessary energy expenditures or competence-mediated fratricide. As the *pht* genes are highly expressed at T2, it is tempting to speculate that the negative regulation of the competence induction by Pht proteins in a transition metal-rich environment until optimal state bacterial cells are achieved before entering the competent state is an important long-term fitness strategy. Alternatively, it was previously reported that competence induction and signal propagation in pneumococcus could be mediated through either the cell-cell contact or the quorum sensing-based diffusion of the CSP. In the direct cell-cell contact study, these authors proposed that a subpopulation of pneumococcal cells (in strain R800) overexpress and transmit CSP to other cells via cell-to-cell collision and that CSP peptides were bound to its cell surface receptor ComD (27). In contrast, another study reported that competence induction (in strain Rx) followed the quorum sensing manner where threshold accumulation of secreted diffusive CSP peptides was required (70). Previously, we have reported that during pneumonia-derived sepsis, the signal of pneumococcal competence was more likely propagated through the cell-cell contact model (26). Perhaps, the removal of four cell surface proteins in the *∆phtABDE* mutant allows for better physical contact between bacteria that leads to hypercompetence, but this observation requires further investigation.

Finally, one unexpected finding in this study is the temporal discrepancy in the onset of the competent state as revealed between IVIS imaging versus RNA-seq. By IVIS imaging, we have recently reported that D39-*ssbB-luc* entered the competent state naturally between 20 and 24 hpi (Fig. 1) (26). However, by both RNA-seq and confirmation by the qRT-PCR, we showed that D39-*ssbB-luc* entered competent state as early as 12 hpi, underscored by the upregulation of “early” competence genes *comCDE*, as well as high levels of expression of the “late” competent genes *cibAB*, *ssbB*, *dprA comGB*, *comGD*, *comGE*, *comGF*, and *comGG*. The expressions of these genes are maintained for the remainder of the infection cycle until the septic mice hit the moribund state, and this is consistent with the live IVIS imaging that showed a persistent and prolonged competent state that propagated systemically throughout the entire mouse after the breach of alveolar-capillary barrier. It should be noted that the competence signals increased significantly when pneumococcal cells entered the systemic circulation, suggesting a much more favorable nutrient-rich environment for sustaining the competent state. Future efforts will characterize in detail both temporal and molecular events that drive the competent development between T1 and T2 by combining RNA-seq and more sensitive live imaging that are coupled with mass genetic knockout analysis of adaptation genes and decipher how they impact the competence induction during pneumonia-derived sepsis.

## MATERIALS AND METHODS

### Bacterial culture and inoculum preparation

The bacterial culture and mouse infection were carried out as previously described (26). Bacterial cultures were grown statically in a 37°C incubator with 5% CO_2_ unless otherwise stated. Briefly, a fresh colony of D39-*ssbB-luc* strain (AD2502) grown on Todd-Hewitt Agar was cultured in Todd-Hewitt medium supplemented with 5% yeast extract (THY) broth (Becton Dickinson, Franklin Lakes, NJ, USA) until optical density at 600 nm (OD_600_) of 0.2. The broth culture of exponentially growing pneumococci was then spread onto the Columbia agar with 5% sheep blood (R01217; Thermo Scientific, Waltham, MA, USA) to circumvent features found in planktonic growth such as long-chain formation. The bacteria were incubated for 5 hours at 37°C before harvest, washed thrice with saline, and diluted to the desired concentration. Bacterial cells were centrifuged at 3,000 × *g* for 5 min.

### Construction of firefly luc reporter strain

Firefly *luc* gene was fused downstream to the desired pneumococcal genes to generate reporter strains. Pneumococcal firefly luc reporter strains were constructed as previously described (26). In short, the amplicon of competence-specific *ssbB* gene was inserted into the pEVP3-derived plasmid (30) harboring the firefly luc gene (pEVP3-*luc*) via BamHI/KpnI enzyme digestion and ligation. The resulting pEVP3-*luc-ssbB* plasmid was transformed into the recipient pneumococcal strains, and the chloramphenicol-resistant transformants were selected.

### Mouse infection

Male CD-1 mice (7–8 weeks old) were purchased from Charles River Laboratories (Boston, MA, USA). All animals were housed in positively ventilated microisolator cages within a room equipped with automatic recirculating water and laminar, high-efficiency particle accumulation filter system. Animals were acclimated upon arrival and provided with autoclaved food, water, and beddings throughout the experiments. Mice (groups of five) were anesthetized with isoflurane prior to intranasal administration of pneumococcal inoculum. The inoculum dose was validated by serial dilution plating on THY agar plates with or without appropriate marker antibiotics. Mice were euthanized at four time points (T1 = 0 hpi, T2 = 12 hpi, T3 = 24 hpi, and T4 = 40–44 hpi), and lungs were collected promptly for total RNA extraction. The harvested lungs were sliced into smaller pieces and stored at −80°C. For T1 (0 hpi), mice were sacrificed within 10 minutes of inoculation with D39-*ssbB-luc*.

### *In vivo* mice imaging

Live imaging of mice was performed by using an IVIS SpectrumCT imaging system (Perkin-Elmer, Waltham, MA, USA). Mice were anesthetized with 3% isoflurane in an induction chamber followed by intraperitoneal injection of d-luciferin potassium 100 mg kg^−1^ (LUCK; GoldBio, St. Louis, MO, USA) dissolved in DPBS (21031CV; Corning, Corning, NY, USA) for 10 minutes to allow uniform spread of the substrate prior to imaging. Luminescence images were captured with the following settings: binning factor = 8, f number = 1, field of view = 25.4 cm, and luminescent exposure time = 60 s. The acquired images were analyzed by Living Image Software (Perkin-Elmer).

### Lung tissue processing and RNA isolation

Frozen lung slices were placed into a −20°C chilled mortar and homogenized with −20°C chilled pestle. Liquid nitrogen was added periodically during grinding prior to the signs of tissue softening and thawing. After the tissues were ground into smaller pieces, 1.0 mL of TRIzol and 1.0 g of aluminum oxide powder were added, and samples were subjected to continuous homogenization for an additional 5 minutes. Liquid nitrogen was again added periodically, as the frozen slurry provides the best grinding efficiency. After grinding, the slurry was collected and centrifuged at 500 × *g* for 30 seconds to remove the aluminum oxide. Then, 0.15-mm zirconium oxide beads were added to the supernatant for bead beating for 10 minutes to further lyse any residual bacteria. The final RNA was extracted and purified from TRIzol following the manufacturer’s instructions.

### Library preparation, sequencing, and RNA-seq analyses

Construction of libraries and sequencing on the Illumina NovaSeq 6000 were performed at the Roy J. Carver Biotechnology Center at the University of Illinois at Urbana-Champaign. The total RNA isolated above was quantitated by Qubit (Thermo Fisher, MA, USA) and checked for integrity using the Agilent Bioanalyzer 2100 (Agilent, CA, USA). Host and bacterial ribosomal RNAs were removed from 1 µg of total RNA using the Ribo-Zero Epidemiology Kit (Illumina, CA, USA). The rRNA-depleted RNAs were converted into individually barcoded RNAseq libraries with the TruSeq Stranded Total RNA Sample Prep Kit. The individual libraries were barcoded with Unique Dual Indexes that have been developed to prevent index switching. The adaptor-ligated double-stranded cDNAs were amplified by PCR for 8 cycles with the Kapa HiFi Polymerase (Roche, IN, USA). The final libraries were quantitated on Qubit, and the average size was determined on the AATI Fragment Analyzer (Agilent, CA, USA). Finally, the libraries were pooled and sequenced on NovaSeq 6000 S1 lane. The libraries were sequenced from one end of the fragments for a total of 100 bp. The quality of the raw FASTQ reads was evaluated by FastQC (http://www.bioinformatics.babraham.ac.uk/projects/fastqc), followed by adaptor removal and quality trimming by Trimmomatic v0.39 (71). The high-quality reads (at least 70% of the original sequence length with an average Phred quality score of ≥25) were mapped against the completed genome of *S. pneumoniae* reference strain D39 (NCBI accession NC_008533) using STAR RNA-seq aligner v2.7.10a with the default parameters (72). Gene feature counts were summarized using featureCounts from the Subread package v2.0.3 (73). Differential expression analysis was performed using the DESeq2 v1.36.0 Bioconductor package with default parameters (74), which utilizes merged raw counts (from featureCounts) as an input and creates a table of differentially expressed genes with important columns, such as gene ID, log_2_ fold change, *P* value, and adjusted *P* value. Genes with log_2_ fold change of ≥1 (twofold change) and adjusted *P* value for multiple comparisons of ≤0.05 were considered as being differentially expressed in our analysis. Multiple testing correction was performed using the Benjamini-Hochberg false discovery rate method. The hierarchical clustering analysis was performed to summarize the gene expression data using pheatmap R functions. Gene ontology term and Kyoto Encyclopedia of Genes and Genomes pathway enrichment analyses of the differentially expressed genes were conducted to gain insight into their functions and the pathways they fall into, using clusterProfiler v3.10 (75). The raw FASTQ reads have been submitted to the NCBI’s Sequence Read Archive (SRA) under the BioSample ID SAMN35715190–SAMN35715209. The detailed gene expression profile with log_2_ fold changes and *P*_adj_ values of pneumococcal genes across each time point can be referred to the Excel file uploaded as File S2.

### Construction of pneumococcal mutant strains

Pneumococcal mutant strains were generated by gene replacement. The antibiotic resistance gene was cloned from the Janus cassette and spliced with the sequences flanking the target gene in the parental genome. Q5 high-fidelity DNA polymerase (New England Biolabs) was used for all PCR amplifications conducted. Flanking sequences were annealed to the antibiotic resistance gene by using the NEBuilder HiFi DNA assembly master mix (New England Biolabs, Ipswich, MA, USA). The assembled template was then amplified by using the nested primers, and the resulting amplicon was purified by either PCR and/or gel purification kits (K0701, K0692; Thermo Scientific, Waltham, MA, USA). The purified product was added into a pneumococcal culture grown in THY broth until OD_600_ of 0.2 along with exogenous CSP1 (100 ng mL^−1^) for artificial induction of pneumococcal competence for homologous recombination. Detailed procedures for molecular genetic manipulations are provided in the Supplemental Information.

### *In vitro* luc assays

*S. pneumoniae* strain CP1250 and its isogenic derivatives harboring the *luc* gene fused downstream to the competence-specific *ssbB* gene were cultured in 96-well plate for competence induction studies. C + Y_A_ (61) and C + Y_B_ (62) broth media of two different pH (6.8 or 7.8) values were used to control the susceptibility to natural competence induction. For transition metal supplementation in C + Y_B_, 432 µg L^−1^ of CuSO_4_, FeSO_4_•7H_2_O, or ZnSO_4_•7H_2_O was added either individually or in combination. Culture conditions were maintained throughout the entire experimental procedures. d-Luciferin potassium (GoldBio) was added to each well to a final concentration of 0.65 mM. For artificial induction of the competent state, exogenous CSP1 was added to a final concentration of 100 ng mL^−1^. The Wallac Victor two multilabel counter (Perkin-Elmer) was maintained at 37°C to measure both bacterial growth and relative luminescence unit emission.

### *In vitro* genetic transformation studies

Transformation studies were performed as previously described (24, 25, 39). *S. pneumoniae* strains were cultured in C + Y_A/B_, and CSP1 was added to a final concentration of 100 ng mL^−1^ when the OD_600_ reached 0.2. Purified genomic DNA-harboring streptomycin resistance *rpsL* gene was added to the recipient cultures to a final concentration of 1,000 ng mL^−1^ to allow transformation via homologous recombination. Transformation mixtures were allowed to rest in 37°C incubator with 5% CO_2_ for 1 hour, after which the cells were serially diluted and plated on THY agar plates with or without streptomycin for enumeration.

### Statistical analyses

Statistical analyses presented in this study were performed by the GraphPad Prism statistical software package. The quantitative data are presented as the mean ± standard deviation. Statistical significance was expressed as *P* ≤ 0.05, *P* ≤ 0.01, *P* ≤ 0.001, *P* ≤ 0.0001, or ns (not significant).

## SUPPLEMENTAL MATERIAL

File S1 spectrum.03050-23-s0001.docx Supplemental methods, tables, and figures.

File S2 spectrum.03050-23-s0002.xls Pneumococcal gene expression during pnuemonia-derived sepsis.

ASM does not own the copyrights to Supplemental Material that may be linked to, or accessed through, an article. The authors have granted ASM a non-exclusive, world-wide license to publish the Supplemental Material files. Please contact the corresponding author directly for reuse.
