# Supplementary material for: Time-resolved RNA-seq analysis to unravel the in vivo competence induction by Streptococcus pneumoniae during pneumonia-derived sepsis
Source: Microbiol Spectr. 2024 Feb 2;12(3):e03050-23. doi: 10.1128/spectrum.03050-23 (PMC10913500; doi:10.1128/spectrum.03050-23)
Supplement: File S1 — Supplemental methods, tables, and figures. [file spectrum.03050-23-s0001.docx]

**SUPPLEMENTARY INFORMATION**

**Time-resolved RNA-Seq analysis to unravel the *in vivo* competence induction by *Streptococcus pneumoniae* during pneumonia-derived sepsis**

Myung Whan Oh^a ꝉ^, Jingjun Lin^a,b ꝉ^, Sook Yin Chong^a^, Shi Qian Lew^a^, Tauqeer Alam^a*^, Gee W Lau^a*^

^a^Department of Pathobiology, University of Illinois at Urbana-Champaign, Urbana, Illinois, 61802.

^b^Current address: Department of Medical Microbiology and Immunology, University of California, Davis, CA 95616.

^ꝉ^These authors contributed equally to this work.

Running title: RNA-Seq of pneumococcal competence in lung infection

^*^To whom correspondence may be addressed. Tauqeer Alam, mtalam@illinois.edu and Gee W. Lau, geelau@illinois.edu; Address: 2001 South Lincoln Ave, Urbana, IL 61802.

**This PDF file includes:**

Supplementary Methods

Figures S1 to S2

Tables S1 to S9

Supplementary information references

**Supplementary Methods**

**PCR, gene splicing and cloning**

Genetic manipulation methods including PCR amplification, gene splicing, and cloning for the purpose of generating mutant strains used in this study (Table S1) were as previously described.(1, 2) Q5 DNA polymerase was used for PCR amplifications following manufacturer’s instruction (M0491, New England Biolabs). Gene splicing was performed using the NEBuilder^®^ HiFi DNA Assembly Master Mix (E2621, New England Biolabs).

**Construction of the Δ*pht* mutants**

Upstream and downstream fragments of the wild-type *Streptococcus pneumoniae* (pneumococcus) strain D39(3, 4) *pht* genes were amplified by primer set PhtX UF/PhtX UR and PhtX DF/PhtX DR using the genomic DNA of D39 wildtype strain as the template (Table S2). The amplicon of kanamycin resistance gene was generated by PCR amplification using the KanF/KanR. The three amplified fragments were assembled and amplified using the nested primer set PhtX Fx/PhtX Rx, and the resulting PCR product was used to transform the parental strains (D39 and CP1250) to generate the respective mutants. To generate the *ssbB-luc* reporter mutant strains, D39 *ssbB-luc* and CP1250 *ssbB-luc* strains were transformed with the previously described nested PCR products.

**Construction of pneumococcal firefly luciferase reporter strains.**

Pneumococcal firefly luciferase reporter strains were generated as previously described.(1, 2) An amplicon of *ssbB* gene was inserted into the pEVP3-derived plasmid containing the firefly luciferase (pEVP3-*luc*) with BamHI/KpnI digestion followed by ligation. The resulting plasmid, pEVP3 *ssbB-luc*, was transformed into the recipient pneumococcal strains D39 and CP1250 by selecting for chloramphenicol-resistant transformants, as confirmed by PCR amplification.

**
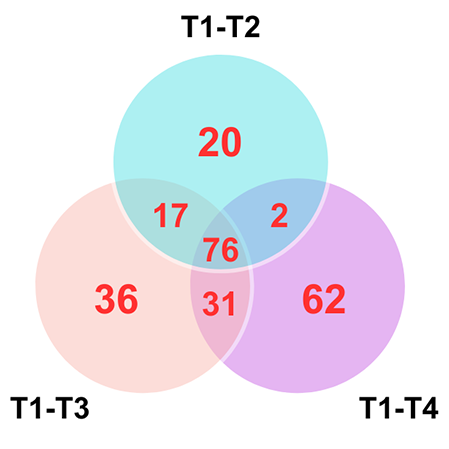
**

**Figure S1.** A Venn diagram displaying number of downregulated genes across each timepoint. In total, the expression of 20, 36 and 62 genes were uniquely downregulated at T2, T3 and T4 respectively, while numbers in the overlapping regions indicate downregulated genes at two or all of three timepoints.

**
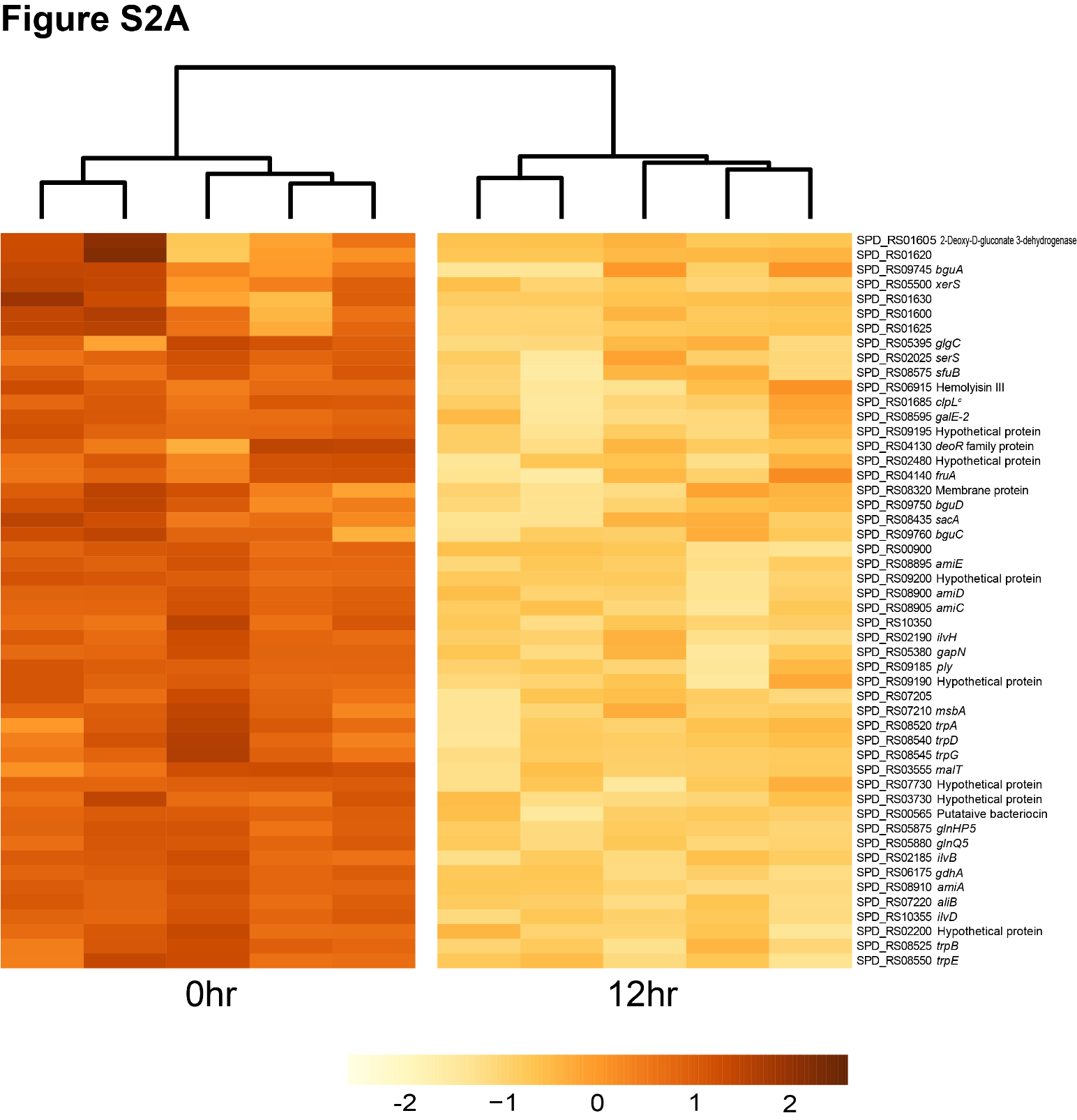
**

**
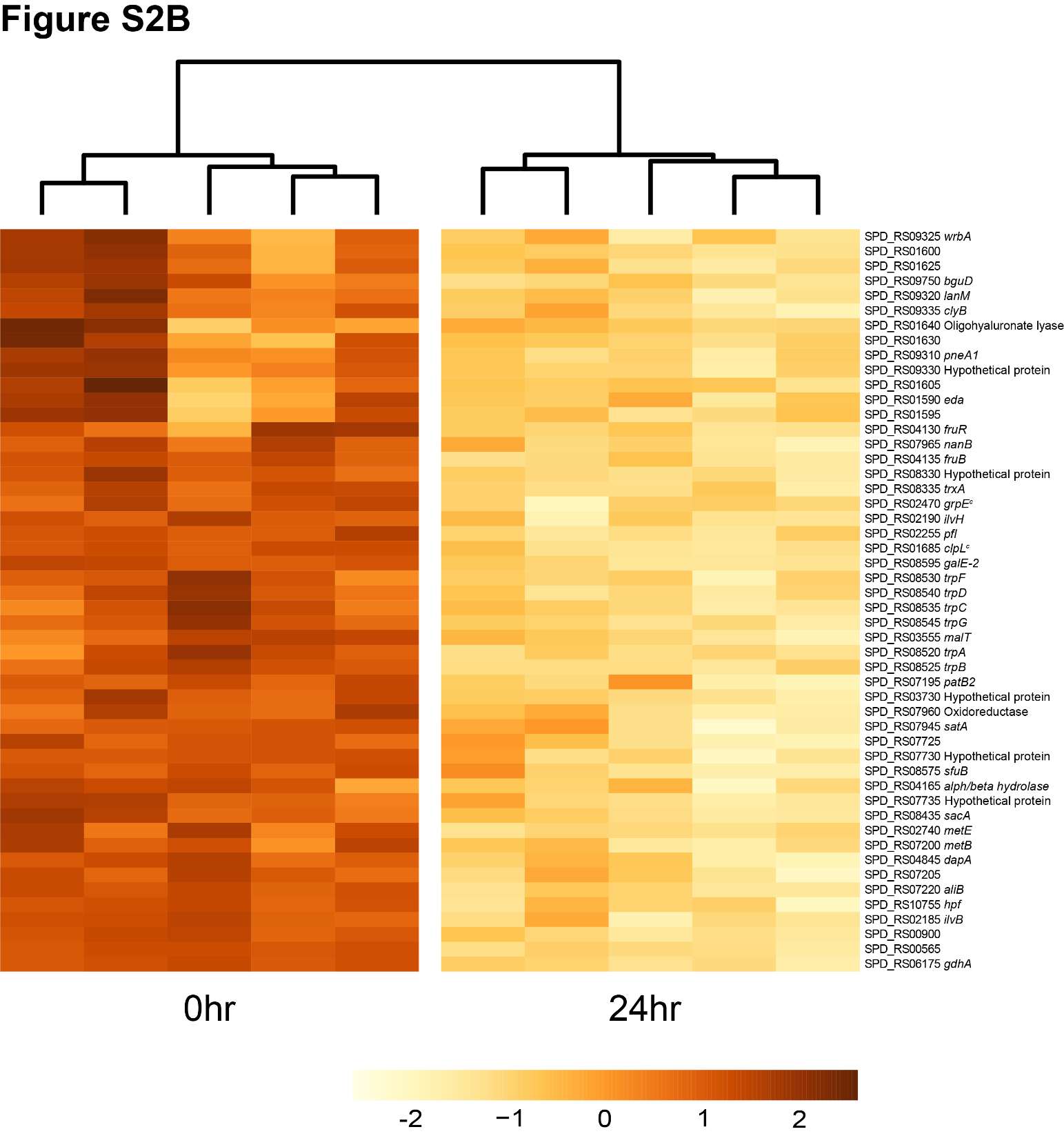
**

**
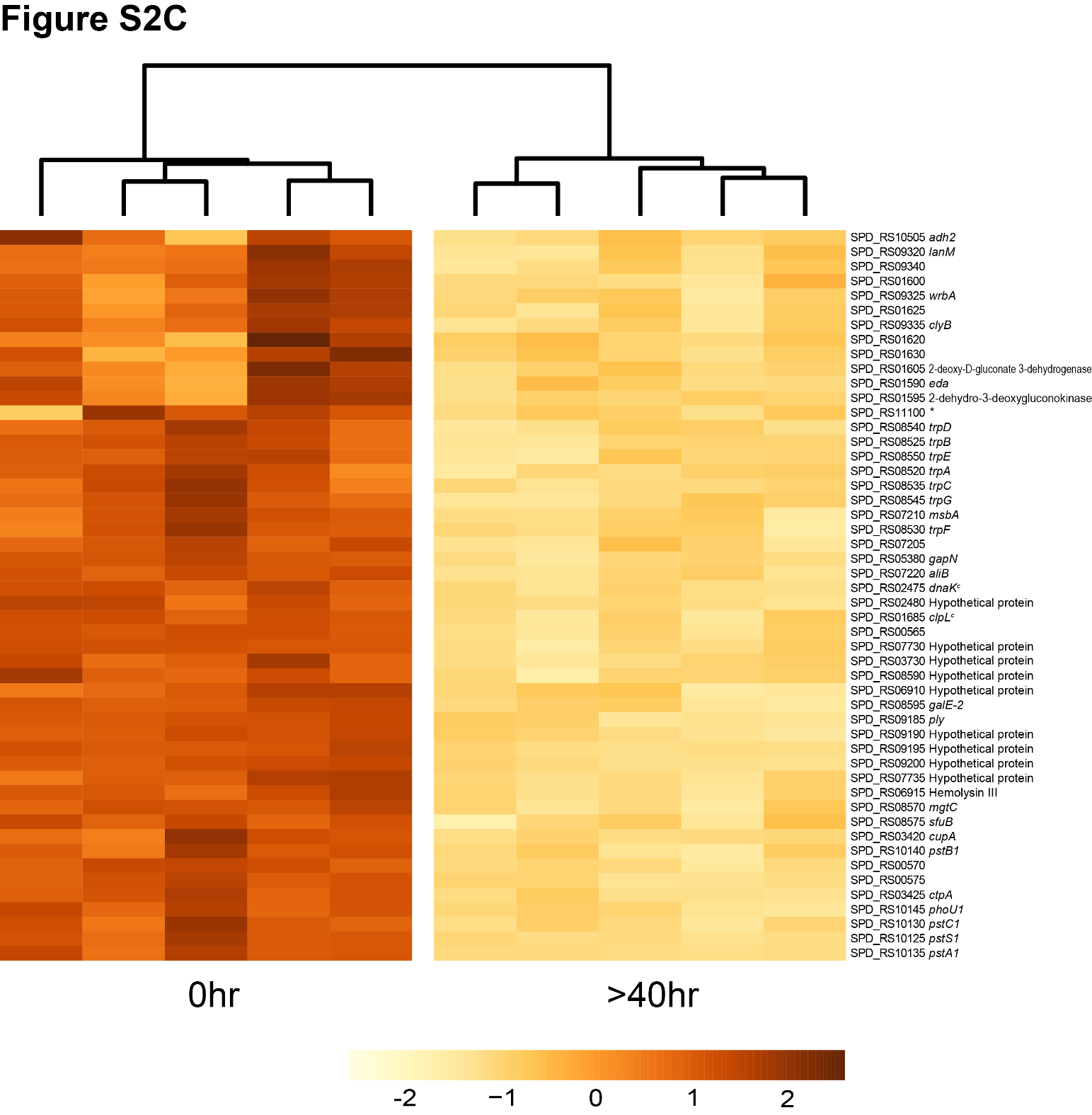
**

**Figure S2.** Heatmaps displaying differentially expressed pneumococcal genes recovered from the infected lungs. Each heatmap showcases up to 50 genes above the *p_adj_* cutoff value that were downregulated between the indicated timepoints. Scale bar indicates the gene expression level based on the log_2_ fold change value. The superscripts indicate early, late or delayed competence genes (a: early, b: late, c: delayed). Asterisk (*) indicates the unannotated locus tags. Blank genes are shown in Table S3.

**Table S1.** *Streptococcus pneumoniae* strains used in this study.

| **Strain** | **Relevant Properties** | **Origin/Reference** |
| --- | --- | --- |
| D39 (WT) | Wild-type *S. pneumoniae,* serotype 2 | References (3, 4) |
| D39 *ssbB-luc* | D39 with a promoter-less firefly luciferase (*luc*) reporter gene transcriptionally fused downstream to the *ssbB* gene; Cm^R^ | References (1, 2) |
| D39 ∆*phtA* | D39 with ∆*phtA*::Kan, Kan^R^ | This work |
| D39 ∆*phtB* | D39 with ∆*phtB*::Kan, Kan^R^ | This work |
| D39 ∆*phtD* | D39 with ∆*phtD*::Kan, Kan^R^ | This work |
| D39 ∆*phtE* | D39 with ∆*phtE*::Kan, Kan^R^ | This work |
| D39 ∆*phtABDE* | D39 with ∆*phtD*::Kan, ∆*phtABE*::Erm; Kan^R^, Erm^R^ | This work |
| CP1250 | Non-encapsulated Rx-derivative strain | Reference(5) |
| CP1250 *ssbB-luc* | CP1250 with a promoter-less firefly luciferase (*luc*) reporter gene transcriptionally fused downstream to the *ssbB* gene; Cm^R^ |  |
| CP1250 ∆*phtA-ssbB-luc* | CP1250 with ∆*phtA*::Kan and a promoter-less firefly luciferase (*luc*) reporter gene transcriptionally fused downstream to the *ssbB* gene; Kan^R^, Cm^R^ | This work |
| CP1250 ∆*phtB-ssbB-luc* | CP1250 with ∆*phtB*::Kan and a promoter-less firefly luciferase (*luc*) reporter gene transcriptionally fused downstream to the *ssbB* gene; Kan^R^, Cm^R^ | This work |
| CP1250 ∆*phtD-ssbB-luc* | CP1250 with ∆*phtD*::Kan and a promoter-less firefly luciferase (*luc*) reporter gene transcriptionally fused downstream to the *ssbB* gene; Kan^R^, Cm^R^ | This work |
| CP1250 ∆*phtE-ssbB-luc* | CP1250 with ∆*phtE*::Kan and a promoter-less firefly luciferase (*luc*) reporter gene transcriptionally fused downstream to the *ssbB* gene; Kan^R^, Cm^R^ | This work |

**Table S2.** PCR amplification Oligonucleotide primers for used in this study.

| Primer name | Sequence (5’-3’) | Used in |
| --- | --- | --- |
| PhtA UF | TCTATCACTAGATGGGAGGTCAGTT | D39 ∆*phtA,*  CP1250 *ssbB-luc* ∆*phtA* |
| PhtA UR | ACATTATCCATTAAAAATCAACCTTGCGTTGTTAAAAGGAAGTAATC | D39 ∆*phtA,*  CP1250 *ssbB-luc* ∆*phtA* |
| PhtA DF | GTTTTAGTACCTAGGGCCCAGTCCCAACTCGTAAGAACAAACAC | D39 ∆*phtA,*  CP1250 *ssbB-luc* ∆*phtA* |
| PhtA DR | GATTATCAACCCAACAGATGAACAA | D39 ∆*phtA,*  CP1250 *ssbB-luc* ∆*phtA* |
| PhtA Fx | TCATCTGTGGTGTAGCGTCCCTGTG | D39 ∆*phtA,*  CP1250 *ssbB-luc* ∆*phtA* |
| PhtA Rx | ACAAACGACTTGATCCAATATACAATG | D39 ∆*phtA,*  CP1250 *ssbB-luc* ∆*phtA* |
| PhtB UF | ACTTGTCGTTAAAGAAGAAGGATTG | D39 ∆*phtB,*  CP1250 *ssbB-luc* ∆*phtB* |
| PhtB UR | ACATTATCCATTAAAAATCAAACAAGTAGATAGTCTCTTGGCTTTGTTA | D39 ∆*phtB,*  CP1250 *ssbB-luc* ∆*phtB* |
| PhtB DF | GTTTTAGTACCTAGGGCCCGCAAACCTAGTTCATAAGCACAG | D39 ∆*phtB,*  CP1250 *ssbB-luc* ∆*phtB* |
| PhtB DR | CTTGAAAGTAAGTTATCAAAACAAGAGAG | D39 ∆*phtB,*  CP1250 *ssbB-luc* ∆*phtB* |
| PhtB Fx | ATGTTCTTGTTTTTGACGATTGATT | D39 ∆*phtB,*  CP1250 *ssbB-luc* ∆*phtB* |
| PhtB Rx | TTGTTTGCGACGATTAAGTACTAC | D39 ∆*phtB,*  CP1250 *ssbB-luc* ∆*phtB* |
| PhtD UF | GCTTATGGTCTGATTCGCTTAC | D39 ∆*phtD,*  CP1250 *ssbB-luc* ∆*phtD* |
| PhtD UR | ACATTATCCATTAAAAATCAAACTTGATTTTCATTCTTTCCTCACTTT | D39 ∆*phtD,*  CP1250 *ssbB-luc* ∆*phtD* |
| PhtD DF | GTTTTAGTACCTAGGGCCCGCTGAAAAACTATTGGCTTTATTAAGG | D39 ∆*phtD,*  CP1250 *ssbB-luc* ∆*phtD* |
| PhtD DR | TTAGAAGTTGGGTCAAAGCCAC | D39 ∆*phtD,*  CP1250 *ssbB-luc* ∆*phtD* |
| PhtD Fx | ATCCAAATCTAAAAAAATCCAAAGT | D39 ∆*phtD,*  CP1250 *ssbB-luc* ∆*phtD* |
| PhtD Rx | CTCCTACATAAGTAATCGGAACAT | D39 ∆*phtD,*  CP1250 *ssbB-luc* ∆*phtD* |
| PhtE UF | GTAATCAATCAACTAACTTTTATTTTTTTC | D39 ∆*phtE,*  CP1250 *ssbB-luc* ∆*phtE* |
| PhtE UR | ACATTATCCATTAAAAATCAAACTTTATCTGATCTCATAGCGTAAG | D39 ∆*phtE,*  CP1250 *ssbB-luc* ∆*phtE* |
| PhtE DF | GTTTTAGTACCTAGGGCCCCACATAGACTCAAGGATACGATAAC | D39 ∆*phtE,*  CP1250 *ssbB-luc* ∆*phtE* |
| PhtE DR | AGTGATAAAGTCAAGTTAGTGGATG | D39 ∆*phtE,*  CP1250 *ssbB-luc* ∆*phtE* |
| PhtE Fx | TAAACATAATACTTTCCATCTCCCT | D39 ∆*phtE,*  CP1250 *ssbB-luc* ∆*phtE* |
| PhtE Rx | CTCACCCTGAATCTGATGAAAAAG | D39 ∆*phtE,*  CP1250 *ssbB-luc* ∆*phtE* |
| ErmF | CCGGGCCCAAAATTTGTTTGATTT | D39 ∆*phtABDE* |
| ErmR | AGTCGGCAGCGACTCATAGAAT | D39 ∆*phtABDE* |
| KanF | GTTTGATTTTTAATGGATAATGT | D39 ∆*phtA*, D39 ∆*phtB,*  D39 ∆*phtD,* D39 ∆*phtE,*  D39 ∆*phtABDE,*  CP1250 ∆*phtA-ssbB-luc*,  CP1250 ∆*phtB-ssbB-luc,*  CP1250 ∆*phtD-ssbB-luc,*  CP1250 ∆*phtE-ssbB-luc,* |
| KanR | GGGCCCTAGGTACTAAAACAA | D39 ∆*phtA*, D39 ∆*phtB,*  D39 ∆*phtD,* D39 ∆*phtE,*  D39 ∆*phtABDE,*  CP1250 ∆*phtA-ssbB-luc*,  CP1250 ∆*phtB-ssbB-luc,*  CP1250 ∆*phtD-ssbB-luc,*  CP1250 ∆*phtE-ssbB-luc,* |
| ComA qPCR F | CAGGTGGTCAACGTCAGAGA | qPCR validation for *comA* gene |
| ComA qPCR R | CAGCAATAGTCAAGCGGTGA | qPCR validation for *comA* gene |
| ComB qPCR F | AATCGGCAACCTCATCAGTC | qPCR validation for *comB* gene |
| ComB qPCR R | CCTCGCCCTGAGACTTGTAG | qPCR validation for *comB* gene |
| ComC qPCR F | CAACCTCATCTCCCCACCT | qPCR validation for *comC* gene |
| ComC qPCR R | CAGTTTGTAGCTTTGAAGGAAAAAG | qPCR validation for *comC* gene |
| ComD qPCR F | CACCGTCACAACGAAAAAGA | qPCR validation for *comD* gene |
| ComD qPCR R | TCGATGGATGAAACAGTATGAGA | qPCR validation for *comD* gene |
| CibA qPCR F | AGCTGCCAAACCAGAACCTA | qPCR validation for *cibA* gene |
| CibA qPCR R | AGATATTGATGGCGGTCTCG | qPCR validation for *cibA* gene |
| DprA qPCR F | GAACGCATCTTAGCCTCTGC | qPCR validation for *dprA* gene |
| DprA qPCR R | TCATTTTCCTGCCCGTAATC | qPCR validation for *dprA* gene |
| ComGC qPCR F | GGCTTTCCACCACCTTAACA | qPCR validation for *comGC* gene |
| ComGC qPCR R | ATCAGCGTGCTTTTCTTGCT | qPCR validation for *comGC* gene |
| CbpD qPCR F | GTAGCCATCCACCGTCGTAT | qPCR validation for *cbpD* gene |
| CbpD qPCR R | CTGGTGAAATGCAGACAGGA | qPCR validation for *cbpD* gene |
| ClpL qPCR F | GTCGTAACCGTGCTGGTTTT | qPCR validation for *clpL* gene |
| ClpL qPCR R | AGCAAGCTCCGTCTTACCAA | qPCR validation for *clpL* gene |
| HtrA qPCR F | GTTTCGCAATTCCTGCAAAT | qPCR validation for *htrA* gene |
| HtrA qPCR R | CCGAACGAACAATTACACCA | qPCR validation for *htrA* gene |
| GyrA qPCR F | CCATTTTGGATAGCGAGCAT | qPCR validation for *gyrA* gene |
| GyrA qPCR R | GGAAAAACGCATTGAGGGTA | qPCR validation for *gyrA* gene |
| PhtA qPCR F | GTCGGTTGTGGACTTGGTTG | qPCR validation for *phtA* gene |
| PhtA qPCR R | CAGCGCAAATCACAAGTCGA | qPCR validation for *phtA* gene |
| PhtB qPCR F | AACTGCCTTTGAGTCAACGC | qPCR validation for *phtB* gene |
| PhtB qPCR R | GGCGGTTCGACTTGTGATTT | qPCR validation for *phtB* gene |
| PhtD qPCR F | TCTAGCAGGTTCAGTGGCAG | qPCR validation for *phtD* gene |
| PhtD qPCR R | TTGACCAGCCTGATCACCAT | qPCR validation for *phtD* gene |
| PhtE qPCR F | ATGATTCACCTAGCGCCCAA | qPCR validation for *phtE* gene |
| PhtE qPCR R | AGTTCCACTGATAGGCACCC | qPCR validation for *phtE* gene |

**Table S3. Genes in the heatmaps (Figure 2A and Figure S2) with blank annotations.**

| Timepoint | Up/Down regulation | NC_008533.2  Locus tag | CP000410.2  Locus tag | Description |
| --- | --- | --- | --- | --- |
| T1-T2 | **Up** | SPD_RS00615 | SPD_0115 | HlyD family secretion protein, degenerate transporter |
|  |  | SPD_RS03090 | SPD_0572 | Thiol:disulfide oxidoreductase., surface-exposed thioredoxin-family lipoprotein |
|  |  | SPD_RS08475 | SPD_1589 | Small integral membrane protein |
|  |  | SPD_RS09860 | SPD_1856 | S-adenosylmethionine-dependent methyltransferase, degenerate |
|  |  | SPD_RS10420 | SPD_1969 | Hexosaminidase/ Glycosyl hydrolase-related protein |
| T1-T3 | **Up** | SPD_RS00615 | SPD_0115 | HlyD family secretion protein, degenerate transporter |
|  |  | SPD_RS02125 |  | CPBP family intramembrane glutamic endopeptidase |
|  |  | SPD_RS06765 | SPD_1266 | Transmembrane component of energizing module of putative ECF transporter |
|  |  | SPD_RS08045 | SPD_1514 | ABC transporter, ATP-binding protein |
|  |  | SPD_RS09945 | SPD_1874 | Surface immunogenic protein, LysM domain-containing protein |
| T1-T4 | **Up** | SPD_RS00470 | SPD_0088 | Multiple sugar ABC transporter, membrane-spanning permease protein |
|  |  | SPD_RS00475 | SPD_0089 | ABC transporter permease protein |
|  |  | SPD_RS00485 | SPD_0090 | ABC transporter substrate-binding protein |
|  |  | SPD_RS00615 | SPD_0115 | HlyD family secretion protein, degenerate transporter |
|  |  | SPD_RS08045 | SPD_1514 | ABC transporter, ATP-binding protein |
|  |  | SPD_RS08770 | SPD_1645 | Transcriptional regulator, MarR family |
|  |  | SPD_RS09940 | SPD_1872 | Transcriptional regulator, MarR family, degenerate |
|  |  | SPD_RS09945 | SPD_1874 | Surface immunogenic protein, LysM domain-containing protein |
|  |  | SPD_RS10665 | SPD_2016 | tRNA dihydrouridine synthase B |
| T1-T2 | **Down** | SPD_RS01620 | SPD_0295 | PTS system mannose/fructose/N-acetylgalactosamine-transporter subunit IIB |
|  |  | SPD_RS01630 | SPD_0297 | PTS system mannose/fructose/sorbose family transporter subunit IID |
|  |  | SPD_RS01600 | SPD_0291 | RpiB/LacA/LacB family sugar-phosphate isomerase |
|  |  | SPD_RS01625 | SPD_0296 | PTS mannose/fructose/sorbose/N-acetylgalactosamine transporter subunit IIC |
|  |  | SPD_RS00900 | SPD_0161 | NRAMP family divalent metal transporter |
|  |  | SPD_RS10350 | SPD_1954 | metal-sulfur cluster assembly factor |
|  |  | SPD_RS07205 |  | ATP-binding cassette domain-containing protein |
| T1-T3 | **Down** | SPD_RS01600 | SPD_0291 | RpiB/LacA/LacB family sugar-phosphate isomerase |
|  |  | SPD_RS01625 | SPD_0296 | PTS mannose/fructose/sorbose/N-acetylgalactosamine transporter subunit IIC |
|  |  | SPD_RS01630 | SPD_0297 | PTS system mannose/fructose/sorbose family transporter subunit IID |
|  |  | SPD_RS01605 | SPD_0292 | gluconate 5-dehydrogenase |
|  |  | SPD_RS01595 | SPD_0290 | PfkB family carbohydrate kinase |
|  |  | SPD_RS07205 |  | ATP-binding cassette domain-containing protein |
|  |  | SPD_RS00900 | SPD_0161 | NRAMP family divalent metal transporter |
|  |  | SPD_RS00565 | SPD_0106 | lactococcin 972 family bacteriocin |
| T1-T4 | **Down** | SPD_RS09340 | SPD_1753 | S8 family serine peptidase |
|  |  | SPD_RS01600 | SPD_0291 | RpiB/LacA/LacB family sugar-phosphate isomerase |
|  |  | SPD_RS01625 | SPD_0296 | PTS mannose/fructose/sorbose/N-acetylgalactosamine transporter subunit IIC |
|  |  | SPD_RS01620 | SPD_0295 | PTS system mannose/fructose/N-acetylgalactosamine-transporter subunit IIB |
|  |  | SPD_RS01630 | SPD_0297 | PTS system mannose/fructose/sorbose family transporter subunit IID |
|  |  | SPD_RS07205 | NA | ATP-binding cassette domain-containing protein |
|  |  | SPD_RS00565 | SPD_0106 | lactococcin 972 family bacteriocin |
|  |  | SPD_RS00900 | SPD_0161 | NRAMP family divalent metal transporter |
|  |  | SPD_RS00570 | SPD_0107 | bacteriocin-associated integral membrane family protein |
|  |  | SPD_RS00575 | SPD_0108 | ABC transporter ATP-binding protein |

**Table S4. List of genes represented by the gene ontology symbols indicating the functional annotation of differentially expressed genes in Figure 3.**

|  | **Descriptions** | **Gene ID** |
| --- | --- | --- |
| T1-T2 | Alpha-amino acid biosynthetic process | *serS/ilvB/ilvN/ilvC/metF/asd/dapA/glyA/trpA/trpB/trpF/trpC/trpD/trpE/ilvD* |
|  | Cellular biogenic amine metabolic process | *trpA/trpB/trpF/trpC/trpD/trpE* |
|  | Amine metabolic process | *trpA/trpB/trpF/trpC/trpD/trpE* |
|  | Amine biosynthetic process | *trpA/trpB/trpF/trpC/trpD/trpE* |
|  | Cellular biogenic amine biosynthetic process | *trpA/trpB/trpF/trpC/trpD/trpE* |
|  | Cellular amine metabolic process | *trpA/trpB/trpF/trpC/trpD/trpE* |
|  | Cellular amino acid biosynthetic process | *serS/ilvB/ilvN/ilvC/metF/asd/dapA/glyA/SPD_RS08025/trpA/trpB/trpF/trpC/trpD/trpE/ilvD* |
|  | Alpha-amino acid metabolic process | *serS/ilvB/ilvN/ilvC/metF/asd/dapA/glyA/SPD_RS07200/trpA/trpB/trpF/trpC/trpD/trpG/trpE/ilvD* |
|  | Aromatic amino acid family metabolic process | *SPD_RS08025/trpA/trpB/trpF/trpC/trpD/trpE* |
|  | Aromatic amino acid family biosynthetic process | *SPD_RS08025/trpA/trpB/trpF/trpC/trpD/trpE* |
|  | Organic substance transport | *SPD_RS00335/SPD_RS00350/SPD_RS01620/SPD_RS01625/SPD_RS01630/aliA/brnQ/livM/exp5/SPD_RS04140/SPD_RS05875/SPD_RS05880/aliB/amiF/amiE/amiA/secE/SPD_RS09750/SPD_RS09755/SPD_RS09760/cglC/malC* |
|  | Branched-chain amino acid metabolic process | *ilvB/ilvN/ilvC/asd/ilvD* |
|  | Branched-chain amino acid biosynthetic process | *ilvB/ilvN/ilvC/asd/ilvD* |
| T1-T3 | Transport | *comA/SPD_RS00580/SPD_RS00900/SPD_RS01625/SPD_RS01630/aliA/SPD_RS02010/brnQ/SPD_RS03425/livH/exp5/SPD_RS04140/SPD_RS05145/SPD_RS05875/SPD_RS05880/SPD_RS06235/SPD_RS06365/potD/aliB/SPD_RS07575/SPD_RS07935/SPD_RS07940/SPD_RS07950/SPD_RS07970/SPD_RS07975/SPD_RS08315/SPD_RS08450/SPD_RS08575/SPD_RS08580/SPD_RS08645/pbuX/SPD_RS08795/SPD_RS08800/SPD_RS08810/SPD_RS08875/amiF/amiE/amiD/amiC/amiA/slgT/SPD_RS09750/SPD_RS09755/SPD_RS09760/cglC/SPD_RS09955/pstC/pstA/pstB/phoU/malX/malC/malD* |
|  | Localization | *comA/SPD_RS00580/SPD_RS00900/SPD_RS01625/SPD_RS01630/aliA/SPD_RS02010/brnQ/SPD_RS03425/livH/exp5/SPD_RS04140/SPD_RS05145/SPD_RS05875/SPD_RS05880/SPD_RS06235/SPD_RS06365/potD/aliB/SPD_RS07575/SPD_RS07935/SPD_RS07940/SPD_RS07950/SPD_RS07970/SPD_RS07975/SPD_RS08315/SPD_RS08450/SPD_RS08575/SPD_RS08580/SPD_RS08645/pbuX/SPD_RS08795/SPD_RS08800/SPD_RS08810/SPD_RS08875/amiF/amiE/amiD/amiC/amiA/slgT/SPD_RS09750/SPD_RS09755/SPD_RS09760/cglC/SPD_RS09955/pstC/pstA/pstB/phoU/malX/malC/malD* |
|  | Establishment of localization | *comA/SPD_RS00580/SPD_RS00900/SPD_RS01625/SPD_RS01630/aliA/SPD_RS02010/brnQ/SPD_RS03425/livH/exp5/SPD_RS04140/SPD_RS05145/SPD_RS05875/SPD_RS05880/SPD_RS06235/SPD_RS06365/potD/aliB/SPD_RS07575/SPD_RS07935/SPD_RS07940/SPD_RS07950/SPD_RS07970/SPD_RS07975/SPD_RS08315/SPD_RS08450/SPD_RS08575/SPD_RS08580/SPD_RS08645/pbuX/SPD_RS08795/SPD_RS08800/SPD_RS08810/SPD_RS08875/amiF/amiE/amiD/amiC/amiA/slgT/SPD_RS09750/SPD_RS09755/SPD_RS09760/cglC/SPD_RS09955/pstC/pstA/pstB/phoU/malX/malC/malD* |
|  | Transmembrane transport | *comA/SPD_RS00580/aliA/SPD_RS02010/SPD_RS03425/livH/exp5/SPD_RS04140/SPD_RS05145/SPD_RS05875/SPD_RS05880/SPD_RS06235/SPD_RS06365/aliB/SPD_RS07575/SPD_RS07935/SPD_RS07940/SPD_RS07950/SPD_RS07970/SPD_RS07975/SPD_RS08315/SPD_RS08450/SPD_RS08575/SPD_RS08580/SPD_RS08645/pbuX/SPD_RS08795/SPD_RS08800/SPD_RS08875/amiD/amiC/amiA/slgT/SPD_RS09750/SPD_RS09755/cglC/SPD_RS09955/pstC/pstA/pstB/malX/malC/malD* |
|  | Alpha-amino acid metabolic process | *purF/serS/ilvB/ilvN/ilvC/ilvA/glnA/metE/metF/asd/dapA/SPD_RS05940/SPD_RS07200/trpA/trpB/trpF/trpC/trpD/trpG/trpE/SPD_RS10070/ilvD* |
|  | Alpha-amino acid biosynthetic process | *serS/ilvB/ilvN/ilvC/ilvA/glnA/metE/metF/asd/dapA/SPD_RS05940/trpA/trpB/trpF/trpC/trpD/trpE/ilvD* |
|  | Branched-chain amino acid metabolic process | *ilvB/ilvN/ilvC/ilvA/asd/SPD_RS05940/ilvD* |
|  | Branched-chain amino acid biosynthetic process | *ilvB/ilvN/ilvC/ilvA/asd/SPD_RS05940/ilvD* |
|  | Multi-organism process | *SPD_RS00735/celB/SPD_RS05990/ply/slgA1/slgA2/cglC/comC1* |
|  | Cellular amino acid biosynthetic process | *serS/ilvB/ilvN/ilvC/ilvA/glnA/metE/metF/asd/dapA/SPD_RS05940/trpA/trpB/trpF/trpC/trpD/trpE/ilvD* |
|  | Cellular biogenic amine metabolic process | *trpA/trpB/trpF/trpC/trpD/trpE* |
|  | Amine metabolic process | *trpA/trpB/trpF/trpC/trpD/trpE* |
|  | Amine biosynthetic process | *trpA/trpB/trpF/trpC/trpD/trpE* |
|  | Cellular biogenic amine biosynthetic process | *trpA/trpB/trpF/trpC/trpD/trpE* |
|  | Cellular amine metabolic process | *trpA/trpB/trpF/trpC/trpD/trpE* |
|  | Carbohydrate metabolic process | *strH/talC/SPD_RS01360/SPD_RS01600/dexB/pflB/lacG-1/SPD_RS04135/nplT/glgB/glgC/glgD/glgA/lacC/zwf/nanE-1/nanB/SPD_RS08435/galE-2/galK/treC/gtfA/SPD_RS09745/malP/SPD_RS10430* |
|  | Multi-organism cellular process | *SPD_RS00735/celB/SPD_RS05990/ply/cglC/comC1* |
|  | Cellular carbohydrate metabolic process | *SPD_RS01600/lacG-1/SPD_RS04135/glgB/glgC/glgD/glgA/lacC/galK/treC/gtfA* |
|  | Cellular amino acid metabolic process | *purF/serS/ilvB/ilvN/ilvC/ilvA/glnA/metE/metF/asd/dapA/SPD_RS05940/gdhA/SPD_RS07200/trpA/trpB/trpF/trpC/trpD/trpG/trpE/SPD_RS10070/ilvD* |
| T1-T4 | Cellular biogenic amine metabolic process | *trpA/trpB/trpF/trpC/trpD/trpE* |
|  | Amine metabolic process | *trpA/trpB/trpF/trpC/trpD/trpE* |
|  | Amine biosynthetic process | *trpA/trpB/trpF/trpC/trpD/trpE* |
|  | Cellular biogenic amine biosynthetic process | *trpA/trpB/trpF/trpC/trpD/trpE* |
|  | Cellular amine metabolic process | *trpA/trpB/trpF/trpC/trpD/trpE* |
|  | Transport | *comA/SPD_RS00470/SPD_RS00475/SPD_RS00900/SPD_RS01610/SPD_RS01620/SPD_RS01625/SPD_RS01630/SPD_RS02290/lacE-1/brnQ/SPD_RS03425/exp5/SPD_RS04140/lmb/SPD_RS05145/SPD_RS05875/SPD_RS05880/SPD_RS06230/SPD_RS06295/SPD_RS07085/aliB/SPD_RS07575/SPD_RS08440/SPD_RS08445/SPD_RS08575/SPD_RS08580/SPD_RS08645/SPD_RS08795/SPD_RS08800/SPD_RS08810/SPD_RS08875/amiF/amiE/amiD/amiC/slgT/secE/SPD_RS09750/SPD_RS09755/SPD_RS09760/cglC/pstC/pstA/pstB/phoU/glpF* |
|  | Localization | *comA/SPD_RS00470/SPD_RS00475/SPD_RS00900/SPD_RS01610/SPD_RS01620/SPD_RS01625/SPD_RS01630/SPD_RS02290/lacE-1/brnQ/SPD_RS03425/exp5/SPD_RS04140/lmb/SPD_RS05145/SPD_RS05875/SPD_RS05880/SPD_RS06230/SPD_RS06295/SPD_RS07085/aliB/SPD_RS07575/SPD_RS08440/SPD_RS08445/SPD_RS08575/SPD_RS08580/SPD_RS08645/SPD_RS08795/SPD_RS08800/SPD_RS08810/SPD_RS08875/amiF/amiE/amiD/amiC/slgT/secE/SPD_RS09750/SPD_RS09755/SPD_RS09760/cglC/pstC/pstA/pstB/phoU/glpF* |
|  | Establishment of localization | *comA/SPD_RS00470/SPD_RS00475/SPD_RS00900/SPD_RS01610/SPD_RS01620/SPD_RS01625/SPD_RS01630/SPD_RS02290/lacE-1/brnQ/SPD_RS03425/exp5/SPD_RS04140/lmb/SPD_RS05145/SPD_RS05875/SPD_RS05880/SPD_RS06230/SPD_RS06295/SPD_RS07085/aliB/SPD_RS07575/SPD_RS08440/SPD_RS08445/SPD_RS08575/SPD_RS08580/SPD_RS08645/SPD_RS08795/SPD_RS08800/SPD_RS08810/SPD_RS08875/amiF/amiE/amiD/amiC/slgT/secE/SPD_RS09750/SPD_RS09755/SPD_RS09760/cglC/pstC/pstA/pstB/phoU/glpF* |

**Table S5. Top 50 upregulated genes shown in the Figure 2A heatmap between T1 (0-hpi) and T2 (12-hpi).**

| NC_008533.2  Locus tag | CP000410.2  Locus tag | Description | Gene name |
| --- | --- | --- | --- |
| SPD_RS00735 | SPD_0132 | Two-peptide bacteriocin peptide CibB | *cibB* |
| SPD_RS09890 | SPD_1862 | Late competence protein ComGB | *comGB* |
| SPD_RS01840 | SPD_0338 | Hypothetical protein |  |
| SPD_RS04455 | SPD_0831 | Replication protein |  |
| SPD_RS04795 | NA^#^ | Pneumococcal histidine triad-type protein |  |
| SPD_RS00675 | SPD_0124 | Hypothetical protein | *rtgZ1* |
| SPD_RS10435 | SPD_1972 | 6-mannosidase |  |
| SPD_RS03085 | SPD_0571 | Cytochrome c-type biogenesis protein CcdA-1 | *ccdA-1* |
| SPD_RS08485 | SPD_1591 | Putative hemin importer |  |
| SPD_RS10765 | SPD_2035 | DNA transporter ATPase, ComF operon protein A | *comFA* |
| SPD_RS02385 | SPD_0444 | Endo-beta-N-acetylglucosaminidase EndoD | *endoD* |
| SPD_RS10640 | SPD_2011 | Glycerol uptake facilitator protein | *glpF* |
| SPD_RS03095 | SPD_0573 | Peptide methionine sulfoxide reductase MsrAB2 | *msrAB2* |
| SPD_RS10430 | SPD_1971 | α-mannosidase |  |
| SPD_RS00355 | SPD_0070 | Galactosamine-6-phosphate isomerase | *agaS* |
| SPD_RS10420 | SPD_1969 | hexosaminidase.2%C Glycosyl hydrolase-related protein |  |
| SPD_RS00430 | SPD_0080 | Cell wall surface anchor family protein PavB | *pavB* |
| SPD_RS08475 | SPD_1589 | Small integral membrane protein |  |
| SPD_RS08470 | SPD_1588 | Putative hemin importer |  |
| SPD_RS08480 | SPD_1590 | General stress protein, Gls24 family, Putative hemin importer | *gls24* |
| SPD_RS00600 | NA | NA |  |
| SPD_RS00615 | SPD_0115 | HlyD family secretion protein, degenerate transporter | *rtgB* |
| SPD_RS00610 | SPD_0114 | Hypothetical protein | *rtgX* |
| SPD_RS00320 | SPD_0063 | Beta-N-acetyl-hexosaminidase | *strH* |
| SPD_RS00605 | NA | NA |  |
| SPD_RS03090 | SPD_0572 | Thiol:disulfide oxidoreductase., surface-exposed thioredoxin-family lipoprotein | *etrx1* |
| SPD_RS04785 | SPD_0890 | Pneumococcal histidine triad protein E | *phtE* |
| SPD_RS05575 | SPD_1038 | Pneumococcal histidine triad protein A | *phtA* |
| SPD_RS00620 | SPD_0116 | putative bacteriocin precursor peptide | *rtgC* |
| SPD_RS08465 | NA | NA |  |
| SPD_RS11035 | NA | NA |  |
| SPD_RS09865 | SPD_1857 | Late competence protein ComGG | *comGG* |
| SPD_RS09870 | SPD_1858 | Late competence protein ComGF | *comGF* |
| SPD_RS08810 | SPD_1652 | Iron compound ABC uptake transporter substrate-binding protein PiuA | *piuA* |
| SPD_RS09875 | SPD_1859 | Late competence protein ComGE | *comGE* |
| SPD_RS05990 | SPD_1122 | DNA protecting protein DprA | *dprA* |
| SPD_RS02925 | SPD_0546 | Branched-chain amino acid transport system carrier protein | *brnQ* |
| SPD_RS04575 | SPD_0850 | Lactoylglutathione lyase | *gloA* |
| SPD_RS08805 | SPD_1651 | Iron compound ABC uptake transporter ATP-binding protein PiuD | *piuD* |
| SPD_RS09860 | NA | methyltransferase domain-containing protein |  |
| SPD_RS08800 | SPD_1650 | Iron compound ABC uptake transporter permease protein PiuC | *piuC* |
| SPD_RS09880 | SPD_1860 | Late competence protein ComGD | *comGD* |
| SPD_RS09110 | SPD_1711 | Single-stranded DNA-binding protein SsbB | *ssbB* |
| SPD_RS10405 | SPD_1965 | Choline-binding protein CbpN | *cbpN* |
| SPD_RS08055 | SPD_1516 | Hypothetical protein |  |
| SPD_RS08060 | SPD_1517 | Hypothetical protein |  |
| SPD_RS08045 | SPD_1514 | ABC transporter, ATP-binding protein |  |
| SPD_RS08050 | SPD_1515 | Hypothetical protein |  |
| SPD_RS00740 | SPD_0133 | Two-peptide bacteriocin peptide CibA | *cibA* |
| SPD_RS08040 | SPD_1513 | Hypothetical protein |  |

^*^The order of the genes is the same as the heatmap shown in the Figure 2A.

^#^Not available

**Table S6. Top 50 upregulated genes shown in the Figure 2A heatmap between T1 (0-hpi) and T3 (24-hpi).**

| NC_008533.2  Locus tag | CP000410.2  Locus tag | Description | Gene name |
| --- | --- | --- | --- |
| SPD_RS04540 | SPD_0844 | Late competence DNA transporter ComEC | *comEC* |
| SPD_RS02115 | SPD_0391 | Biofilm-regulating peptide | *briC* |
| SPD_RS09870 | SPD_1858 | Late competence protein ComGF | *comGF* |
| SPD_RS09865 | SPD_1857 | Late competence protein ComGG | *comGG* |
| SPD_RS09895 | SPD_1863 | Late competence protein ComGA | *comGA* |
| SPD_RS00740 | SPD_0133 | Two-peptide bacteriocin peptide CibA | *cibA* |
| SPD_RS05990 | SPD_1122 | DNA protecting protein DprA | *dprA* |
| SPD_RS02555 | SPD_0474 | Immunity protein BlpZ | *blpZ* |
| SPD_RS09860 | NA | methyltransferase domain-containing protein |  |
| SPD_RS09875 | SPD_1859 | Late competence protein ComGE | *comGE* |
| SPD_RS10920 | SPD_2063 | Two-component system response regulator ComE | *comE* |
| SPD_RS10925 | SPD_2064 | Two-component system sensor histidine kinase ComD | *comD* |
| SPD_RS02925 | SPD_0546 | Branched-chain amino acid transport system carrier protein | *brnQ* |
| SPD_RS08810 | SPD_1652 | Iron compound ABC uptake transporter substrate-binding protein PiuA | *piuA* |
| SPD_RS09885 | SPD_1861 | Late competence protein ComGC | *comGC* |
| SPD_RS00110 | SPD_0023 | Competence positive regulator ComW | *comW* |
| SPD_RS10930 | SPD_2065 | Competence-stimulating peptide precursor ComC | *comC* |
| SPD_RS00655 | SPD_0121 | putative bacteriocin precursor peptide | *rtgT* |
| SPD_RS00675 | SPD_0124 | Hypothetical protein | *rtgZ1* |
| SPD_RS02120 | NA | NA |  |
| SPD_RS09945 | SPD_1874 | Surface immunogenic protein, LysM domain-containing protein |  |
| SPD_RS00665 | SPD_0122 | Hypothetical protein | *rtgY1* |
| SPD_RS08805 | SPD_1651 | Iron compound ABC uptake transporter ATP-binding protein PiuD | *piuD* |
| SPD_RS10405 | SPD_1965 | Choline-binding protein CbpN | *cbpN* |
| SPD_RS08040 | SPD_1513 | Hypothetical protein |  |
| SPD_RS08800 | SPD_1650 | Iron compound ABC uptake transporter permease protein PiuC | *piuC* |
| SPD_RS08055 | SPD_1516 | Hypothetical protein |  |
| SPD_RS08060 | SPD_1517 | Hypothetical protein |  |
| SPD_RS08045 | SPD_1514 | ABC transporter, ATP-binding protein |  |
| SPD_RS08050 | SPD_1515 | Hypothetical protein |  |
| SPD_RS00610 | SPD_0114 | Hypothetical protein | *rtgX* |
| SPD_RS00615 | SPD_0115 | HlyD family secretion protein, degenerate transporter | *rtgB* |
| SPD_RS00600 | NA | NA |  |
| SPD_RS00605 | NA | NA |  |
| SPD_RS00235 | SPD_0049 | Competence-stimulating peptide ABC transporter ATP-binding protein ComA | *comA* |
| SPD_RS00240 | SPD_0050 | Competence-stimulating peptide ABC transporter permease protein ComB | *comB* |
| SPD_RS00735 | SPD_0132 | Two-peptide bacteriocin peptide CibB | *cibB* |
| SPD_RS09880 | SPD_1860 | Late competence protein ComGD | *comGD* |
| SPD_RS09890 | SPD_1862 | Late competence protein ComGB | *comGB* |
| SPD_RS08795 | SPD_1649 | Iron compound ABC uptake transporter permease protein PiuB | *piuB* |
| SPD_RS09110 | SPD_1711 | Single-stranded DNA-binding protein SsbB | *ssbB* |
| SPD_RS06765 | SPD_1266 | Transmembrane component of energizing module of putative ECF transporter |  |
| SPD_RS00270 | SPD_0055 | Phosphoribosylglycinamide formyltransferase | *purN* |
| SPD_RS10955 | NA | NA |  |
| SPD_RS02550 | SPD_0473 | Bacteriocin immunity protein BlpY | *blpY* |
| SPD_RS02565 | SPD_0475 | Putative protease | *pncP* |
| SPD_RS00670 | SPD_0123 | putative bacteriocin precursor peptide | *rtgW1* |
| SPD_RS00265 | SPD_0054 | Phosphoribosylformylglycinamidine cyclo-ligase | *purM* |
| SPD_RS00620 | SPD_0116 | putative bacteriocin precursor peptide | *rtgC* |
| SPD_RS02125 | NA | CPBP family intramembrane glutamic endopeptidase |  |

^*^The order of the genes is the same as the heatmap shown in the Figure 2A.

^#^NA - Not available

**Table S7. Top 50 upregulated genes shown in the Figure 2A heatmap between T1 (0-hpi) and T4 (40-44-hpi).**

| NC_008533.2  Locus tag | CP000410.2  Locus tag | Description | Gene name |
| --- | --- | --- | --- |
| SPD_RS10405 | SPD_1965 | Choline-binding protein CbpN | *cbpN* |
| SPD_RS09940 | SPD_1872 | Transcriptional regulator, MarR family |  |
| SPD_RS09945 | SPD_1874 | Surface immunogenic protein, LysM domain-containing protein |  |
| SPD_RS10665 | SPD_2016 | tRNA dihydrouridine synthase B |  |
| SPD_RS08805 | SPD_1651 | Iron compound ABC uptake transporter ATP-binding protein PiuD | *piuD* |
| SPD_RS02925 | SPD_0546 | Branched-chain amino acid transport system carrier protein | *brnQ* |
| SPD_RS08800 | SPD_1650 | Iron compound ABC uptake transporter permease protein PiuC | *piuC* |
| SPD_RS08810 | SPD_1652 | Iron compound ABC uptake transporter substrate-binding protein PiuA | *piuA* |
| SPD_RS09935 | SPD_1871 | Hypothetical protein |  |
| SPD_RS00605 |  |  |  |
| SPD_RS00615 | SPD_0115 | HlyD family secretion protein, degenerate transporter | *rtgB* |
| SPD_RS00610 | SPD_0114 | Hypothetical protein | *rtgX* |
| SPD_RS00620 | SPD_0116 | putative bacteriocin precursor peptide | *rtgC* |
| SPD_RS00600 | NA | NA |  |
| SPD_RS10955 | NA | NA |  |
| SPD_RS02565 | SPD_0475 | Putative protease | *pncP* |
| SPD_RS08055 | SPD_1516 | Hypothetical protein |  |
| SPD_RS08045 | SPD_1514 | ABC transporter, ATP-binding protein |  |
| SPD_RS08050 | SPD_1515 | Hypothetical protein |  |
| SPD_RS02115 | SPD_0391 | Biofilm-regulating peptide | *briC* |
| SPD_RS08040 | SPD_1513 | Hypothetical protein |  |
| SPD_RS08060 | SPD_1517 | Hypothetical protein |  |
| SPD_RS02550 | SPD_0473 | Bacteriocin immunity protein BlpY | *blpY* |
| SPD_RS02555 | SPD_0474 | Immunity protein BlpZ | *blpZ* |
| SPD_RS06345 | SPD_1187 | LSU ribosomal protein L7/L12 (P1/P2) | *rplL* |
| SPD_RS04570 | SPD_0849 | LSU ribosomal protein L20p | *rplT* |
| SPD_RS04575 | SPD_0850 | Lactoylglutathione lyase | *gloA* |
| SPD_RS06350 | SPD_1188 | LSU ribosomal protein L10p (P0) | *rplJ* |
| SPD_RS02120 | NA | NA |  |
| SPD_RS10005 | SPD_1886 | tRNA-Ser-UGA | *tRNA-Ser-4* |
| SPD_RS08770 | SPD_1645 | Transcriptional regulator, MarR family |  |
| SPD_RS00470 | SPD_0088 | Multiple sugar ABC transporter, membrane-spanning permease protein |  |
| SPD_RS00475 | SPD_0089 | ABC transporter permease protein |  |
| SPD_RS00785 | SPD_0140 | ABC transporter ATP-binding protein |  |
| SPD_RS10645 | SPD_2012 | Alpha-glycerophosphate oxidase | *glpO* |
| SPD_RS00430 | SPD_0080 | Cell wall surface anchor family protein PavB | *pavB* |
| SPD_RS10635 | SPD_2010 | Hypothetical protein |  |
| SPD_RS08795 | SPD_1649 | Iron compound ABC uptake transporter permease protein PiuB | *piuB* |
| SPD_RS10640 | SPD_2011 | Glycerol uptake facilitator protein | *glpF* |
| SPD_RS00235 | SPD_0049 | Competence-stimulating peptide ABC transporter ATP-binding protein ComA | *comA* |
| SPD_RS10650 | SPD_2013 | Glycerol kinase | *glpK* |
| SPD_RS09895 | SPD_1863 | Late competence protein ComGA | *comGA* |
| SPD_RS09110 | SPD_1711 | Single-stranded DNA-binding protein SsbB | *ssbB* |
| SPD_RS09865 | SPD_1857 | Late competence protein ComGG | *comGG* |
| SPD_RS09870 | SPD_1858 | Late competence protein ComGF | *comGF* |
| SPD_RS09885 | SPD_1861 | Late competence protein ComGC | *comGC* |
| SPD_RS00740 | SPD_0133 | Two-peptide bacteriocin peptide CibA | *cibA* |
| SPD_RS09860 | NA | methyltransferase domain-containing protein |  |
| SPD_RS09880 | SPD_1860 | Late competence protein ComGD | *comGD* |
| SPD_RS09890 | SPD_1862 | Late competence protein ComGB | *comGB* |

^*^The order of the genes is the same as the heatmap shown in the Figure 2A.

^#^NA - Not available

**Table S8. Genes included in Figure 5 with the linear fold change in gene expression relative to T1 (0 hpi) across each timepoint with standard error (SE) values.**

| NC_008533.2  Locus tag | CP000410.2  Locus tag | Description | Gene name | T2 FC (SE) | T3 FC (SE) | T4 FC (SE) |
| --- | --- | --- | --- | --- | --- | --- |
| SPD_RS01345 | SPD_0247 | 6-phospho-β-glucosidase | *bglA* | 2.4764 (1.45) | 2.2486 (1.44) | 2.5090 (1.40) |
| SPD_RS02705 | SPD_0503 | 6-phospho- β -glucosidase | *bglA2* | 1.2859 (1.29) | 1.3633 (1.44) | 1.2257 (1.37) |
| SPD_RS02305 | SPD_0427 | 6-phospho- β -galactosidase | *lacG1* | 0.8732 (1.56) | 0.3147 (1.59) | 0.4116 (1.46) |
| SPD_RS05615 | SPD_1046 | 6-phospho- β -galactosidase | *lacG2* | 1.2672 (1.56) | 0.7796 (1.55) | 1.5270 (1.42) |
| SPD_RS01515 | SPD_0277 | 6-phospho- β -glucosidase | *celA* | 0.9246 (1.66) | 0.5067 (1.66) | 1.0966 (1.54) |
| SPD_RS09745 | SPD_1830 | 6-phospho- β -glucosidase | *bguA* | 0.1910 (1.48) | 0.3056 (1.43) | 0.2652 (1.37) |
| SPD_RS01645 | SPD_0301 | Transcriptional regulator RegR | *regR* | 1.4554 (1.18) | 1.1758 (1.17) | 1.0625 (1.13) |
| SPD_RS00235 | SPD_0049 | Competence-stimulating peptide ABC transporter ATP-binding protein ComA | *comA* | 2.0106 (1.27) | 11.3988 (1.69) | 4.2718 (1.22) |
| SPD_RS00240 | SPD_0050 | Competence-stimulating peptide ABC transporter permease protein ComB | *comB* | 1.1457 (1.34) | 9.4340 (1.27) | 3.5165 (1.26) |
| SPD_RS10930 | SPD_2065 | Competence-stimulating peptide precursor ComC | *comC* | 1.5701 (1.44) | 6.2489 (1.38) | 2.6857 (1.36) |
| SPD_RS10925 | SPD_2064 | Two-component system sensor histidine kinase ComD | *comD* | 0.9608 (1.30) | 7.4418 (1.28) | 3.4028 (1.28) |
| SPD_RS10920 | SPD_2063 | Two-component system response regulator ComE | *comE* | 1.1420 (1.29) | 7.6261 (1.27) | 3.2730 (1.26) |
| SPD_RS00110 | SPD_0023 | Competence positive regulator ComW | *comW* | 0.9134 (1.55) | 6.5166 (1.40) | 1.8683 (1.38) |
| SPD_RS00740 | SPD_0133 | Two-peptide bacteriocin peptide CibA | *cibA* | 4.7187 (1.61) | 22.8526 (1.55) | 5.8829 (1.54) |
| SPD_RS05990 | SPD_1122 | DNA protecting protein DprA | *dprA* | 5.0742 (1.44) | 8.1636 (1.41) | 2.5390 (1.39) |
| SPD_RS10730 | SPD_2028 | Choline-binding protein D | *cbpD* | 3.4516 (1.148) | 2.3792 (1.47) | 2.6800 (1.41) |
| SPD_RS09885 | SPD_1861 | Late competence protein ComGC | *comGC* | 4.1111 (1.70) | 5.6643 (1.67) | 4.4707 (1.62) |
| SPD_RS03775 | SPD_0701 | Two-component system response regulator CiaR | *ciaR* | 1.1594 (1.22) | 1.2534 (1.20) | 0.8887 (1.15) |
| SPD_RS03780 | SPD_0702 | Two-component system sensor histidine kinase CiaH | *ciaH* | 1.2575 (1.26) | 1.1694 (1.26) | 0.8994 (1.24) |
| SPD_RS10945 | SPD_2068 | Serine protease, DegP/HtrA | *htrA* | 1.8418 (1.30) | 1.3862 (1.30) | 0.7943 (1.28) |
| SPD_RS02540 | SPD_0472 | Peptide ABC transporter ATP binding/permease protein BlpA | *blpA* | 1.5964 (1.30) | 1.7343 (1.29) | 1.6569 (1.27) |
| SPD_RS02535 | SPD_0471 | Peptide ABC transporter permease protein BlpB | *blpB* | 1.0074 (1.30) | 1.6809 (1.28) | 1.5043 (1.27) |
| SPD_RS02530 | SPD_0470 | Peptide pheromone BlpC | *blpC* | 1.3363 (1.71) | 1.6569 (1.70) | 1.6002 (1.67) |
| SPD_RS02520 | SPD_0468 | Two-component system response regulator BlpR | *blpR* | 0.7646 (1.27) | 0.7601 (1.24) | 0.7177 (1.19) |
| SPD_RS02525 | SPD_0469 | Two-component system sensor histidine kinase BlpH | *blpH* | 0.8259 (1.26) | 0.9073 (1.24) | 0.6371 (1.21) |
| SPD_RS02515 | SPD_0467 | BlpS protein | *blpS* | 0.5177 (1.50) | 1.0460 (1.39) | 0.8507 (1.31) |
| SPD_RS02510 | SPD_0466 | BlpT protein | *blpT* | 1.9908 (1.62) | 2.4224 (1.59) | 3.3007 (1.52) |
| SPD_RS00220 | SPD_0046 | Bacteriocin BlpK | *blpU* | 1.7396 (1.48) | 1.0330 (1.48) | 0.4405 (1.45) |
| SPD_RS02550 | SPD_0473 | Bacteriocin immunity protein BlpY | *blpY* | 1.8973 (1.51) | 4.9679 (1.48) | 4.9042 (1.46) |
| SPD_RS02555 | SPD_0474 | Immunity protein BlpZ | *blpZ* | 3.1972 (1.54) | 7.8115 (1.49) | 4.6977 (1.46) |
| SPD_RS05575 | SPD_1038 | Pneumococcal histidine triad protein A | *phtA* | 6.2048 (1.30) | 1.7068 (1.31) | 0.4650 (1.30) |
| SPD_RS05570 | SPD_1037 | Pneumococcal histidine triad protein B | *phtB* | 2.0777 (1.31) | 0.8910 (1.31) | 0.3048 (1.31) |
| SPD_RS04780 | SPD_0889 | Pneumococcal histidine triad protein D | *phtD* | 2.2309 (1.28) | 0.9990 (1.28) | 0.3241 (1.27) |
| SPD_RS04785 | SPD_0890 | Pneumococcal histidine triad protein E | *phtE* | 4.4916 (1.24) | 1.6845 (1.25) | 0.6564 (1.24) |
| SPD_RS10565 | SPD_1997 | Zinc ABC transporter, substrate-binding lipoprotein AdcA | *adcA* | 2.5033 (1.33) | 2.5003 (1.33) | 1.4473 (1.20) |
| SPD_RS04775 | SPD_0888 | Laminin-binding surface protein | *adcAII* | 1.6931 (1.21) | 0.8336 (1.21) | 0.3380 (1.20) |

**Table S9. Compositions of the C+Y^3,4,5,6^ media used in this study.**

| Media component | C+Y_A_ (6) | C+Y_B_ (7) |
| --- | --- | --- |
| L-asparagine | 39.9 mg/L | none |
| L-cysteine | 20.3 mg/L | none |
| L-glutamine | 20.0 mg/L | none |
| L-tryptophan | 5.47 mg/L | none |
| Arginine | none | 150 mg/L |
| Asparagine | none | 40 mg/L |
| Cystine | none | 120 mg/L |
| Glutamine | none | 20 mg/L |
| Glycine | none | 80 mg/L |
| Isoleucine | none | 200 mg/L |
| Leucine | none | 400 mg/L |
| Serine | none | 250 mg/L |
| Valine | none | 250 mg/L |
| CaCl_2_ | 15.1 mg/L | 2.5 mg/L |
| MgCl_2._6H_2_O | 0.21 g/L | 0.5 g/L |
| MnSO_4_×4H_2_O | 255 μg/L | 25 μg/L |
| CuSO_4_ | 290 μg/L | None |
| FeSO_4_×7H_2_O | 456 μg/L | None |
| ZnSO_4_×7H_2_O | 454 μg/L | None |
| Glucose | 1.82 g/L | None |
| Sucrose | 0.295 g/L | None |
| Maltose | None | 2 g/L |
| Adenosine | 18.2 mg/L | None |
| Adenine | None | 5 mg/L |
| Uridine | 18.2 mg/L | None |
| Sodium acetate | 1.82 g/L | 2 g/L |
| Sodium pyruvate | 273 mg/L | 240 mg/L |
| Biotin | 0.5 mg/L | 0.2 μg/L |
| Nicotinic acid | 0.55 mg/L | 0.2 mg/L |
| Pyridoxine hydrochloride | 0.64 mg/L | 0.2 mg/L |
| D-calcium pantothenate | 1.09 mg/L | 0.6 mg/L |
| Thiamine hydrochloride | 0.58 mg/L | 0.2 mg/L |
| Riboflavin | 0.25 mg/L | 0.1 mg/L |
| Choline chloride | 6.1 mg/L | 4 mg/L |
| Yeast extract | 2.28 g/L | 0.8% (w/v) |
| Bovine serum albumin | 0.729 g/L | 0.48 g/L |
| NaCl | none | 2 g/L |
| K_2_HPO_4_ | 7.75g/L | 6.9 g/L |
| KH_2_PO_4_ | none | 1.34 g/L |
| Casein hydrolysate | 4.56 g/L | none |

**References**

1. Lin J, Lau GW. 2019. DprA-Dependent Exit from the Competent State Regulates Multifaceted Streptococcus pneumoniae Virulence. Infect Immun 87.

2. Lin J, Park P, Li H, Oh MW, Dobrucki IT, Dobrucki W, Lau GW. 2020. Streptococcus pneumoniae Elaborates Persistent and Prolonged Competent State during Pneumonia-Derived Sepsis. Infect Immun 88.

3. Avery OT, Macleod CM, McCarty M. 1944. Studies on the Chemical Nature of the Substance Inducing Transformation of Pneumococcal Types : Induction of Transformation by a Desoxyribonucleic Acid Fraction Isolated from Pneumococcus Type Iii. J Exp Med 79:137-58.

4. Avery OT, MacLeod CM, McCarty M. 1979. Studies on the chemical nature of the substance inducing transformation of pneumococcal types. Inductions of transformation by a desoxyribonucleic acid fraction isolated from pneumococcus type III. J Exp Med 149:297-326.

5. Ravin AW. 1959. Reciprocal capsular transformations of pneumococci. J Bacteriol 77:296-309.

6. Domenech A, Slager J, Veening JW. 2018. Antibiotic-Induced Cell Chaining Triggers Pneumococcal Competence by Reshaping Quorum Sensing to Autocrine-Like Signaling. Cell Rep 25:2390-2400 e3.

7. Stevens KE, Chang D, Zwack EE, Sebert ME. 2011. Competence in Streptococcus pneumoniae is regulated by the rate of ribosomal decoding errors. mBio 2.
